# Epithelia Use Butyrophilin-like Molecules to Shape Organ-Specific γδ T Cell Compartments

**DOI:** 10.1016/j.cell.2016.08.030

**Published:** 2016-09-22

**Authors:** Rafael Di Marco Barros, Natalie A. Roberts, Robin J. Dart, Pierre Vantourout, Anett Jandke, Oliver Nussbaumer, Livija Deban, Sara Cipolat, Rosie Hart, Maria Luisa Iannitto, Adam Laing, Bradley Spencer-Dene, Philip East, Deena Gibbons, Peter M. Irving, Pablo Pereira, Ulrich Steinhoff, Adrian Hayday

**Affiliations:** 1 Francis Crick Institute, London WC2A3LY, UK; 2 Peter Gorer Department of Immunobiology, King’s College London, London SE19RT, UK; 3 MBPhD Programme, University College London, London WC1E 6BT, UK; 4 Department of Gastroenterology, Guy’s and St Thomas’ Foundation Trust, London SE17EH, UK; 5 Department of Immunology, Pasteur Institute, 75015 Paris, France; 6 Institute for Medical Microbiology and Hospital Epidemiology, University of Marburg, 35037 Marburg, Germany

## Abstract

Many body surfaces harbor organ-specific γδ T cell compartments that contribute to tissue integrity. Thus, murine dendritic epidermal T cells (DETCs) uniquely expressing T cell receptor (TCR)-Vγ5 chains protect from cutaneous carcinogens. The DETC repertoire is shaped by *Skint1*, a butyrophilin-like (*Btnl*) gene expressed specifically by thymic epithelial cells and suprabasal keratinocytes. However, the generality of this mechanism has remained opaque, since neither Skint1 nor DETCs are evolutionarily conserved. Here, *Btnl1* expressed by murine enterocytes is shown to shape the local TCR-Vγ7^+^ γδ compartment. Uninfluenced by microbial or food antigens, this activity evokes the developmental selection of TCRαβ^+^ repertoires. Indeed, Btnl1 and Btnl6 jointly induce TCR-dependent responses specifically in intestinal Vγ7^+^ cells. Likewise, human gut epithelial cells express BTNL3 and BTNL8 that jointly induce selective TCR-dependent responses of human colonic Vγ4^+^ cells. Hence, a conserved mechanism emerges whereby epithelia use organ-specific BTNL/Btnl genes to shape local T cell compartments.

## Highlights

• Gut epithelial butyrophilin-like 1 (*Btnl1*) shapes the local γδ T cell compartment
• Other organ-specific epithelial *Btnl* genes select cognate γδ cells in other sites
• Btnl heteromers can specifically activate γδ T cells with cognate T cell receptors
• Human *BTNL* genes reveal a conserved biology of epithelial T cell regulation

## Graphical Abstract

**Figure:**
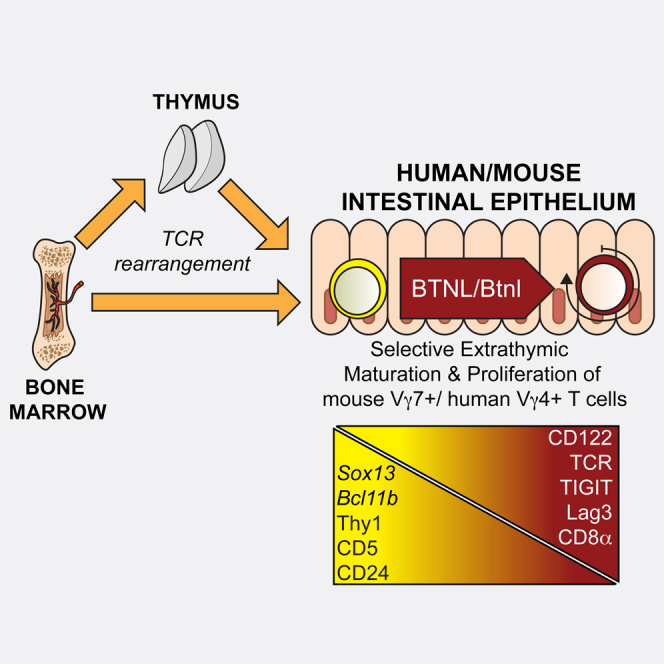

## Teaser

Epithelial cells provide signals that instruct the development and function of their local γδ T cell compartments so that these immune cells can support the non-immune functions of the different barrier tissues.

## Introduction

The specialized differentiation of body surface epithelia is most commonly viewed from the perspective of organ-specific physiological functions, such as nutrient absorption in the gut and prevention of *trans*-epidermal water loss. Likewise, differentiated epithelia provide physical and chemical barriers to pathogens and toxins (Janeway et al., 2001). However, it is now appreciated that body surfaces at steady state comprise diverse cell types, including many immune cells (Vantourout and Hayday, 2013). Among these, intraepithelial lymphocytes (IELs) expressing αβ T cell receptors (TCRs) can mount rapid recall responses to pathogens, while other IELs, commonly expressing γδ TCRs, contribute to the maintenance of body surface integrity that is key to metazoan viability. Thus, murine gut γδ T cells regulate enterocyte differentiation and turnover (Komano et al., 1995) and limit inflammatory damage (Hayday and Tigelaar, 2003, Hayday, 2000, Roberts et al., 1996), while TCRγδ^+^ dendritic epidermal T cells (DETCs) (Kuziel et al., 1987, Stingl et al., 1987a) limit inflammation, promote wound resolution, and increase cutaneous resistance to carcinogens (Girardi et al., 2001, Girardi et al., 2002, Jameson et al., 2002, Strid et al., 2008). Moreover, the skin and gut of jawless fish harbor cells similar to TCRγδ^+^ IELs, arguing that such compartments fulfil critical, evolutionarily conserved roles (Hirano et al., 2013).

In mice, large IEL compartments are defined by γδ TCRs that match particular anatomical sites: Vγ5 in skin, Vγ7 in gut, and Vγ6 in the uterus (Allison and Havran, 1991, Asarnow et al., 1988, Guy-Grand et al., 2013, Itohara et al., 1990, Kyes et al., 1989, Lefrancois and Goodman, 1989, Stingl et al., 1987a, Stingl et al., 1987b, Vantourout and Hayday, 2013). Conceivably, these alignments are determined by organ-specific products of epithelial differentiation, particularly since TCRγδ^+^ IELs are largely unaffected by major histocompatibility complex (MHC) antigens that shape αβ T cell repertoires. Likewise, whereas some γδ TCRs bind CD1 and MR1, genetic studies have largely excluded these MHC-related molecules from the selection of murine γδ cell repertoires (Bigby et al., 1993, Correa et al., 1992, Hayday and Vantourout, 2013, Kuziel et al., 1987, Lefrancois et al., 1990, Pereira et al., 1997).

One insight into how epithelia might shape local IEL repertoires was provided for the skin by the discovery of *Skint1*, the founding member of a novel multi-gene family specifically expressed by thymic epithelial cells and keratinocytes. *Skint1* drives the selective maturation of Vγ5^+^ DETC progenitors, and DETCs are >90% ablated in *Skint1* mutant mice, while all other T cells are unaffected (Barbee et al., 2011, Boyden et al., 2008, Turchinovich and Hayday, 2011). However, the generality of this mechanism for IEL selection was questioned, since neither DETCs nor *Skint1* is broadly conserved and because *Skint* genes are only expressed in skin and thymus (Boyden et al., 2008).

This notwithstanding, *Skint* genes sit within the *Btnl* family comprising six rodent and five human genes. Their poorly understood gene products are structurally similar to CD80 and PDL1 co-stimulatory and inhibitory molecules, which are themselves considered to be evolutionarily related to the MHC (Abeler-Dörner et al., 2012, Afrache et al., 2012, Barbee et al., 2011, Rhodes et al., 2001, Salim et al., 2016, Stammers et al., 2000). By definition, *Btnl*/*BTNL* genes are structurally similar to butyrophilin (*Btn*/*BTN*) genes, of which mice have two and humans six. Butyrophilin genes derive their name (“butter-loving”) from the founding member, *Btn1a1*/*BTN1A1*, that encodes a milk fat micelle-associated protein (Franke et al., 1981). However, this function appears atypical in contrast to the recent implication, albeit largely imprecise, of several BTN/Btn/BTNL/Btnl gene products in immune regulation (Rhodes et al., 2016). Provocatively, human *BTN3A1* facilitates peripheral blood γδ T cell responses to low-molecular-mass microbial and endogenous metabolites (so-called phosphoantigens), although it is not known whether this is mediated by direct TCR-BTN3A1 binding (Adams et al., 2015, Harly et al., 2012, Palakodeti et al., 2012, Vavassori et al., 2013, Wang et al., 2013).

To explore whether *Btnl* genes might mediate epithelial regulation of local γδ T cells, we considered the mouse gut, the major site of *Btnl1*, *Btnl4*, and *Btnl6* expression (Bas et al., 2011). Here, we identify a time window early in the development of young mice in which *Btnl1* expressed by post-mitotic, small intestinal villus epithelial cells critically and selectively promotes the maturation and expansion of Vγ7^+^ T cells, thereby shaping the IEL compartment. Requiring neither microbial nor food antigens, this process evokes *Skint1*-mediated DETC selection and αβ T cell selection by the MHC. Indeed, we show that intestinal epithelial cells expressing Btnl1 jointly with Btnl6 can induce TCR-dependent stimulation uniquely of intestinal Vγ7^+^ T cells.

γδ cells have often been viewed as species specific with few features conserved between mouse and humans (Kazen and Adams, 2011, Vantourout and Hayday, 2013). However, it is increasingly clear that human tissues too harbor large γδ cell compartments with TCRs distinct from those in peripheral blood (Landau et al., 1995, Vantourout and Hayday, 2013; our unpublished data). Furthermore, a large bioinformatics study of thousands of cancer patients presenting with a broad range of carcinomas established that the best correlate of overall survival was a tumor-associated γδ T cell gene signature (Gentles et al., 2015). Hence, there is a pressing need to define how human epithelia interact with tissue-resident γδ T cells.

Addressing this, we provide a refined description of human colonic γδ cells and show that a signature subset expressing TCRVγ4 is specifically regulated by human *BTNL3* and *BTNL8* expressed by human gut epithelium. Hence, the specialized differentiation of intestinal epithelial cells in mice and in humans includes the expression at steady state of site-specific regulators of local T cell compartments.

## Results

### Intestinal Epithelial T Cell Selection

**Figure 1:**
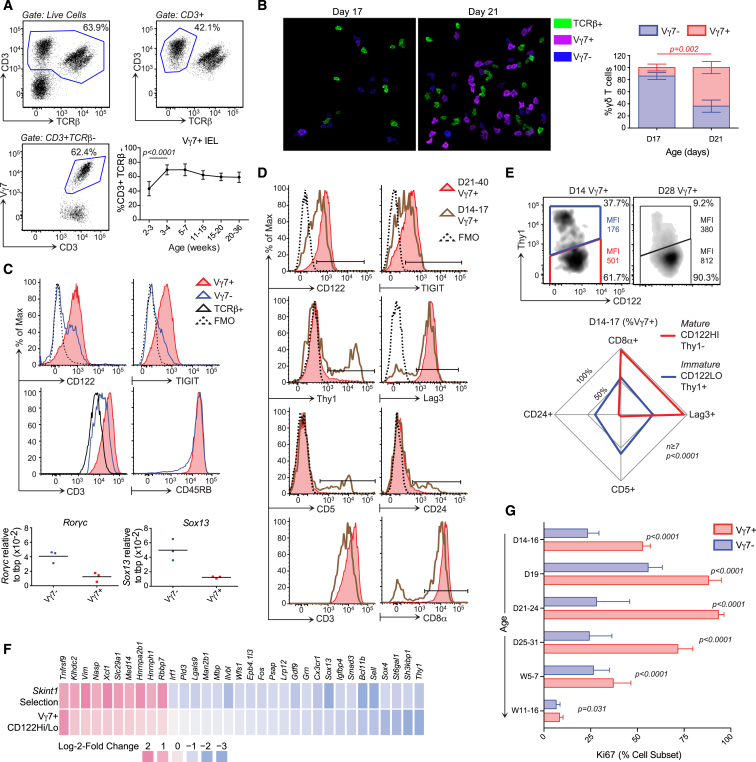
Selective Maturation and Expansion of Intestinal IELs in Mice (A) Gating strategy for small intestinal (SI) Vγ7^+^ IELs in 12-week-old C57Bl/6 mice (n ≥ 12). Bottom right: Vγ7^+^ IEL representation over time (n = 5, week 20–36; n ≥ 12, other time points). (B) IEL composition assessed by confocal microscopy of proximal SI whole mounts (n = *3*) and corresponding quantification (right). (C) Top: surface phenotypes of Vγ7^+^, Vγ7^−^ (CD3^+^TCRβ^−^Vγ7^−^) and αβ IELs from 21- to 40-day-old mice (n ≥ 8). Bottom: gene expression in Vγ7^+^ versus Vγ7^−^ IELs (n = 3). (D) Surface phenotypes of Vγ7^+^ IELs at days 14–17 versus days 21–40 (n ≥ 7). (E) Top: surface phenotype of Vγ7^+^ IELs at day 14 and day 28 (CD122 median fluorescence intensity [MFI]-colored text). Bottom: surface phenotype of CD122^HI^Thy1^−^Vγ7^+^ versus CD122^LO^Thy1^+^Vγ7^+^ IELs at days 14–17 (n ≥ 7). (F) Heatmap of genes differentially expressed between Vγ7^+^CD122^HI^ and Vγ7^+^CD122^LO^ IELs from day 14–17 mice and between *Skint1-*selected and non-selected Vγ5^+^ DETC progenitors (n = 4). (G) Ki67 expression in Vγ7^+^ versus Vγ7^−^ IELs directly ex vivo (n = 4, day 19; n = 8–27, other time points). Data are representative of one (C, qPCR, B and F) or three or more (C, cytometry, D and E, top) independent experiments. Some panels present data pooled from three or more (E), more than ten (G), and >20 (A) independent experiments. D, day; W, week. All error bars represent mean ± SD. See also Figure S1.

**Figure S1:**
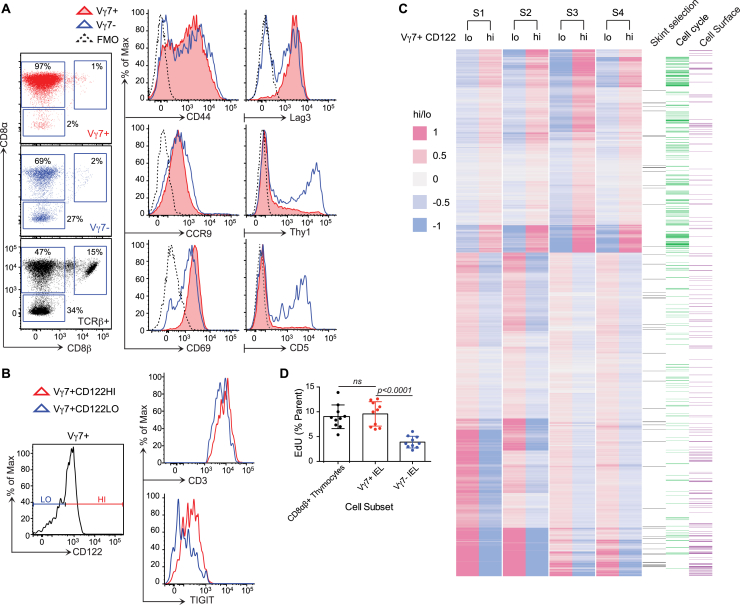
Phenotypic Differences between CD122^HI^ Vγ7^+^ IELs and Other IEL Subsets, Related to Figure 1 (A) Cell surface phenotype of Vγ7^+^, Vγ7^-^ (CD3^+^TCRβ^-^Vγ7^-^) and αβ (TCRβ^+^) IEL from 3-5 week old (W3-5) C57Bl/6 (WT) mice (n ≥ 7). (B) Cell surface phenotype of WT Vγ7^+^CD122^HI^ versus Vγ7^+^CD122^LO^ IEL (n ≥ 7). (C) Heat map of genes differentially expressed (log-2-FoldChange) between Vγ7^+^CD122^hi^ and Vγ7^+^CD122^lo^ IEL sorted from D14-D17 WT mice. Data generated by RNA sequencing (‘cell cycle’ & ‘cell surface’ GO terms annotated). Values scaled to their median value across the samples. (D) 3hr EdU incorporation in vivo in Vγ7^+^ versus Vγ7^-^ IEL from D28 WT mice assessed by flow cytometry in indicated IEL subsets (Vγ7^-^ are CD3^+^TCRβ^-^Vγ7^-^). Data are representative of 1 (C) or ≥ 3 (A,B) independent experiments. Panel (D) presents data pooled from 3 independent experiments. All error bars represent mean ± SD. Related to Figure 1.

By flow cytometry of cells recovered from epithelium, and by confocal visualization of epithelial whole mounts, we found that the signature murine small intestinal Vγ7^+^ IEL compartment largely took shape at 2–3 weeks of age and remained stable for at least 9 months thereafter (Figures 1A and 1B). At day 21, Vγ7^+^ cells mostly phenocopied mature *Skint1*-selected DETCs, expressing uniformly high levels of CD122 (the IL-2R/IL-15Rβ chain), TIGIT (an inhibitory co-receptor), and the TCR (detected with anti-CD3 antibodies) and low levels of RNA for *Rorgc* and *Sox13*, two transcription factors contributing to γδ T cell differentiation (Vantourout and Hayday, 2013) (Figure 1C). Vγ7^−^ IELs (mostly Vγ1 or Vγ4) did not show this phenotype, and whereas both Vγ7^+^ and Vγ7^−^ IEL subsets were mostly CD45RB^hi^, CD44^+^, and CCR9^+^, Vγ7^+^ IELs were distinct in being Lag3^+^, Thy1^−^, CD69^+^, CD5^−^, and CD8αα^+^ (Figures 1C and S1A).

Prior to day 21, however, Vγ7^+^ IELs phenocopied Vγ7^−^ IELs of adult mice. Thus, by sequential gating and radar plots of surface protein co-expression, one could clearly distinguish mature Vγ7^+^ IELs (CD122^hi[MFI > 500]^, Thy1^−^, TIGIT^+^, Lag3^+^, CD8αα^+^, CD5^−^, CD24^−^, TCR^hi^) from putative Vγ7^+^ IEL progenitors (CD122^lo[MFI < 200]^, Thy1^+^, TIGIT^−^, Lag3^−^, CD8αα^−^, CD5^+^, CD24^+^, TCR^lo^) (Figures 1D, 1E, and S1B), with the latter also phenocopying DETC progenitors prior to *Skint*1 selection (Turchinovich and Hayday, 2011).

To further compare IELs with their putative progenitors, CD122^hi^ Vγ7^+^ and CD122^lo^ Vγ7^+^ IELs were purified from the same day 14–17 mice on four independent occasions and assessed by RNA sequencing (RNA-seq) (Figure S1C). Consistent with their distinct phenotypes, the cells showed significantly different expression of many genes for cell surface proteins (Figure S1C). Furthermore, many genes up- (e.g., *Tnfrsf9* [4-1BB/CD137], *Xcl1* [lymphotactin], *Nasp*) or downregulated (e.g., *sox13*, *Bcl11b*, *Cx3cr1*) in CD122^hi^ versus CD122^lo^ Vγ7^+^ cells were likewise regulated by *Skint1* selection of DETC progenitors (Figures 1F and S1C).

Additionally, CD122^hi^ Vγ7^+^ cells were enriched in cell-cycle genes, consistent with which ∼100% of Vγ7^+^ IELs at day 21–24 were Ki67^+^ (i.e., outside of G_0_), compared to <40% of Vγ7^−^ cells (p < 0.0001) (Figure 1G). Likewise, Vγ7^+^ IELs at day 28 phenocopied rapidly dividing thymocytes in that ∼10% incorporated ethynyldeoxyuridine (EdU) (a labeled nucleotide) during a 3-hr pulse, compared to only 4% of Vγ7^−^ IELs (Figure S1D).

In sum, these data are consistent with the gut supporting the selective maturation and expansion of CD122^hi^, Thy1^−^, TIGIT^+^, Lag3^+^, CD8αα^+^, CD5^−^, CD24^−^, TCR^hi^ Vγ7^+^ cells that by weeks 3–4 dominate the γδ IEL compartment. After week 5, the fraction of cycling (Ki67^+^) Vγ7^+^ IELs at steady state declined to levels comparable to Vγ7^−^ IELs (Figure 1G).

### A Gut Epithelial Selecting Element

**Figure 2:**
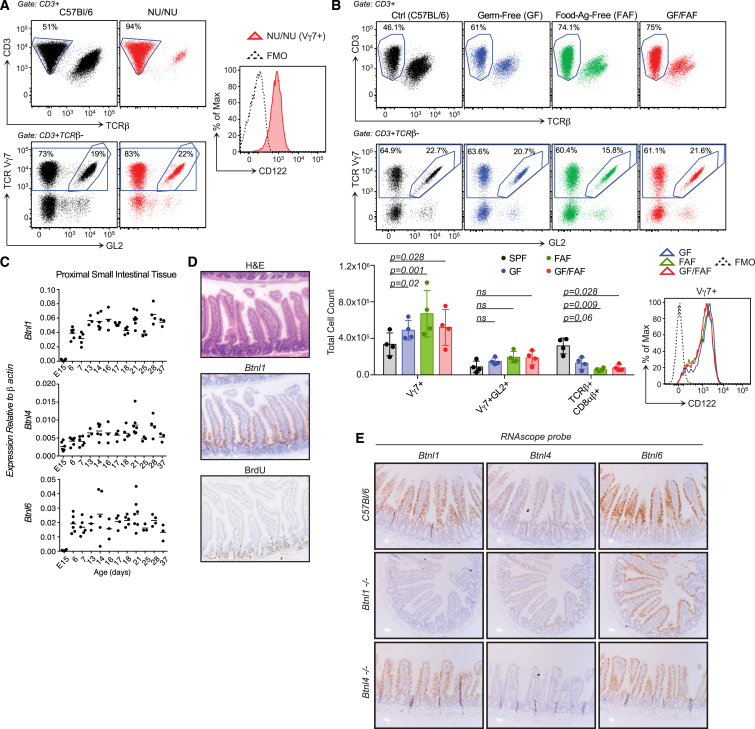
A Gut IEL Selecting Element (A) Left: IEL composition in WT versus NU/NU mice; antibody GL2 detects TRDV2-2-encoded Vδ4 chain. Right: surface CD122 expression on NU/NU Vγ7^+^ IELs (n ≥ 12). (B) IEL composition (top), enumeration (bottom left), and CD122 expression (bottom right) in germ-free (GF), food antigen-free (FAF), or GF-FAF C57Bl/6 mice at weeks 9–13 (n ≥ 4). (C) qRT-PCR of denoted genes. (D) Histological analysis of *Btnl1* RNA (middle: RNAScope) and 3-hr BrdU incorporation in vivo (bottom) in paraffin-embedded SI gut rolls (n ≥ 3). (E) RNAScope of *Btnl1*, *Btnl4*, and *Btnl6* in WT versus *Btnl1*^−/−^ and *Btnl4*^−/−^ mice. Data are representative of two (B), two or more (D and E), or three or more (A) independent experiments. In (C), data are pooled from two independent experiments. All error bars represent mean ± SD. See also Figure S2.

**Figure S2:**
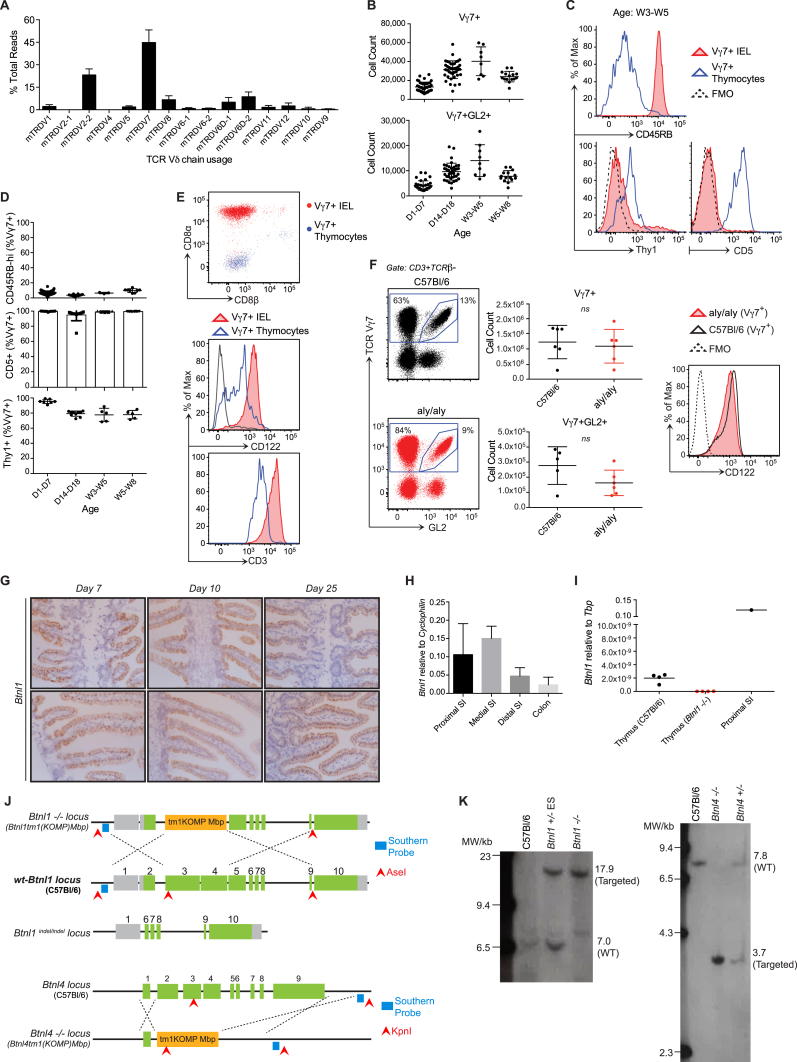
Local Intestinal Development of CD122^HI^ Vγ7^+^ IELs, Related to Figure 2 (A) Deep sequencing of TCR Vδ chain usage in WT Vγ7^+^ IEL sorted from W7-10 C57Bl/6 (WT) mice (n = *3)*. (B) Absolute numbers of WT Vγ7^+^ and Vγ7^+^GL2^+^ thymocytes from WT mice assessed by flow cytometry. C-D) Cell surface phenotype of WT Vγ7^+^ IEL and thymocytes at indicated time points. (E) Cell surface phenotype of Vγ7^+^ thymocytes and IEL isolated from W3-5 WT mice (n = *5*). (F) γδ IEL composition (*left)*, cell count (*middle)* and cell surface CD122 expression (*right*) in WT versus alymphoplasia (a*ly/aly*) mice. (G) Longitudinal RNAscope analysis of *Btnl1* expression during gut development. (H) Gene expression by qRT.PCR along the length of the gut in WT mice (n ≥ *3*). (I) Gene expression by qRT.PCR in the thymus of WT and *Btnl1−/−* animals compared to the proximal small intestine. (J) Organization of WT and targeted loci for *Btnl1−/−, Btnl1*^*indel/indel*^ and *Btnl4*−/− mice. Grey: untranslated region; green: translated region; orange: inserted targeting cassette. Knockout ES cell clones were obtained from the international mouse consortium IKMC-ID 67994 (Btnl1) and 81524 (Btnl4). (K) Southern blot for targeting of alleles in *Btnl1−/−* and *Btnl4−/−* mice. Genomic DNA was digested using the indicated enzymes (arrowheads). Probes targeting the indicated regions were generated to detect the WT and targeted alleles. Data are representative of ≥ 1 (A,K) or ≥ 2 (C,E,G,H,I) independent experiments. Some panels include data pooled from 2 (F), > 3 (D) or > 6 (B) independent experiments. All error bars represent mean ± SD. Related to Figure 2

Because *Skint1* selects for signature Vγ5^+^ DETC progenitors in the thymus, DETCs are absent from athymic NU/NU mice. By contrast, intestinal IELs were present in NU/NU, and although there was some decrease in numbers (average of ∼1.3 × 10^6^ cells compared to >2.0 × 10^6^ cells in euthymic mice; see below), the compartment was again dominated by CD122^hi^ Vγ7^+^ IELs. Moreover, ∼25% of Vγ7^+^ IELs in NU/NU and in euthymic mice reacted with antibody GL2 that detects Vδ4 (TRDV2-2 encoded) chains. Consistent with this, TRDV2-2 sequences accounted for ∼25% of TCRδ chain RNAs expressed by purified Vγ7^+^ IELs (Figures 2A and S2A). In sum, the shaping of the gut Vγ7^+^ IEL compartment did not require a thymus.

Consistent with this, Vγ7^+^ thymocytes were rare, comprising <10% of TCRγδ^+^ cells in fetal and post-natal thymi across the first 8 weeks of life, the peak period of thymus function in mice (Figure S2B). Furthermore, most Vγ7^+^ thymocytes were CD45RB^lo^, Thy1^+^, CD5^hi^, CD122^lo^, TCR^lo^, and CD8αα^−^, thus offering no evidence for intrathymic maturation (Figures S2C–S2E). Likewise, neither lymph nodes nor Peyer’s patches (PP) were required to shape the IEL compartment, since normal numbers of Vγ7^+^ and Vγ7GL2^+^ IELs with signature phenotypes were present in aly/aly (alymphoplasia) mice (Shinkura et al., 1999) that following surgery were confirmed to lack PP and peripheral and mesenteric lymph nodes (MLNs) (Figure S2F).

As intestinal driver(s) of IEL maturation in weanling mice, microbial and/or food antigens were logical candidates. However, C57Bl/6 mice bred into and maintained in a germ-free environment and/or on elemental, protein-antigen-free diet displayed Vγ7^+^ and Vγ7GL2^+^ IEL compartments comparable to conventionally housed counterparts (Figure 2B). The Vγ7^+^ IELs were uniformly TCR^hi^, CD122^hi^, and absolute numbers were somewhat increased, partially compensating for the decline of TCRαβ IELs in germ-free and protein-antigen-free mice (Figure 2B). Thus, the local T cell compartment is most likely shaped by an endogenous intestinal element(s).

In seeking that element(s), we focused on three genes, *Btnl1*, *Btnl4*, and *Btnl6*, that are closely related to *Skint1* (Abeler-Dörner et al., 2012, Afrache et al., 2012, Bas et al., 2011). *Btnl4* was expressed at low levels in proximal small intestine, commencing in the fetus. *Btnl1* and *Btnl6* RNAs were detected at day 6 postpartum, and *Btnl1* levels further increased at around day 14 before the expression of all three *Btnl* genes stabilized (Figure 2C). Expression was in post-mitotic villus enterocytes that are interspersed with IELs and was essentially absent from villus crypts that house replicating epithelial cell progenitors and lack IELs (Figures 2D, 2E, and S2G). *Btnl1* expression peaked in proximal and medial small intestine (Figure S2H), where its expression was >10^7^-fold higher than in the thymus (Figure S2I). These expression patterns could permit *Btnl1*, *Btnl4*, and/or *Btnl6* to act locally upon Vγ7^+^ IELs in weanling mice. To investigate this possibility, we obtained three independent strains of *Btnl1*^−/−^ mice and one of *Btnl4*^−/−^ mice, each generated by targeted mutagenesis of embryonic stem cells (ESCs), and a strain with an internally deleted *Btnl1* locus generated by clustered regularly interspaced short palindromic repeats (CRISPR)-Cas9 in mouse eggs (Figure S2J). The strains were confirmed as gene knockouts by DNA analysis and loss of respective *Btnl* RNAs (Figures 2E and S2I–S2K).

### *Btnl1* Shapes the Intestinal IEL Compartment

**Figure 3:**
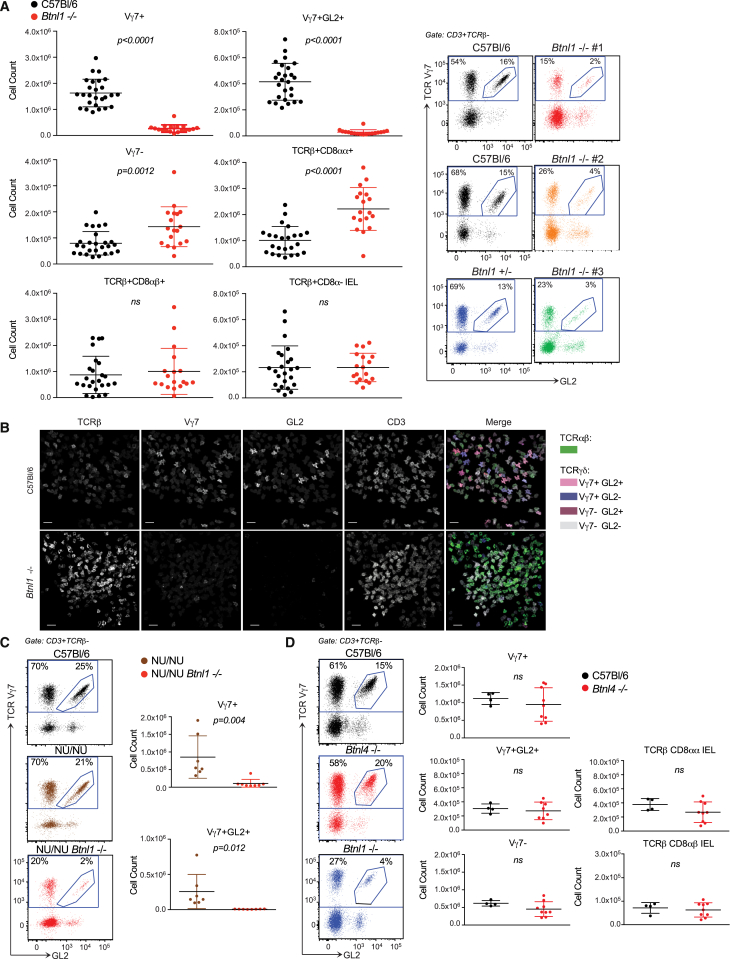
Gut IEL Composition Depends on *Btnl1* (A) Left: enumeration of IEL subsets from week 6–15 WT and *Btnl1*^−/−^ mice by flow cytometry. Right: representative data from three independently derived *Btnl1*^−/−^ lines and controls (n ≥ 6). (B) Confocal microscopy of proximal SI whole mounts from week 10 WT and *Btnl1*^−/−^ mice (n = *3*). (C) Vγ7^+^ IEL representation (left) and enumeration (right) of WT (2.6 m ± 630,000), NU/NU (0.9 m ± 600,000) and *Btnl1*^−/−^ NU/NU mice (0.1 m ± 120,000). (D) Representation (left) and enumeration (right) of IEL subsets from WT, *Btnl1*^−/−^, and *Btnl4*^−/−^ mice. Some panels include data pooled from two (C and D) or six independent (A) experiments. All error bars represent mean ± SD. See also Figure S3.

**Figure S3:**
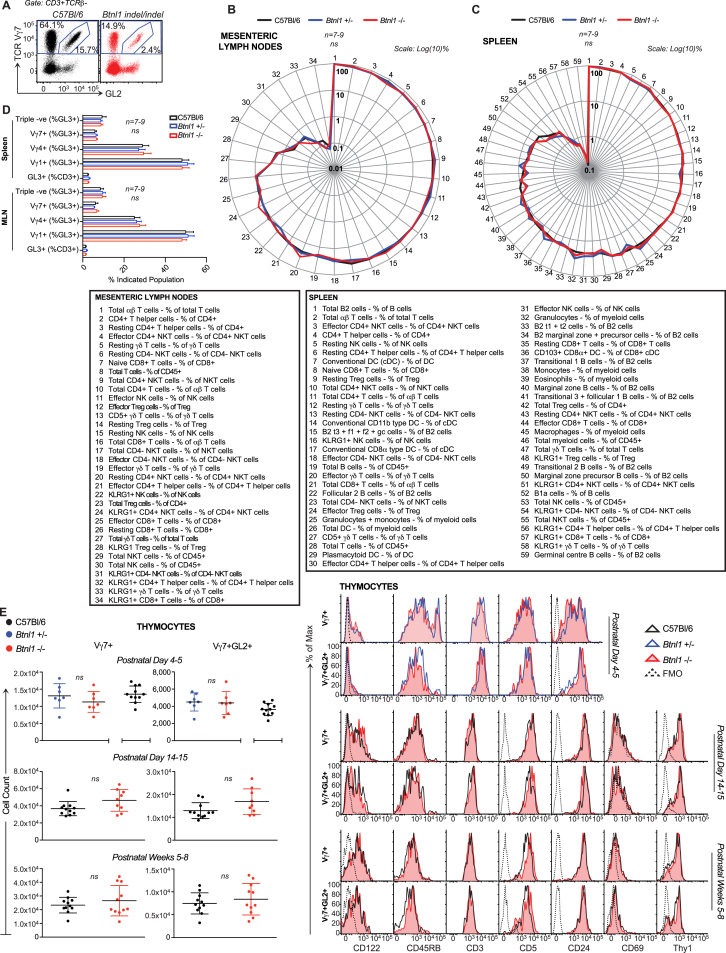
*Btnl1* Has No Detectable Effect on the Systemic T, B, and Myeloid Cell Compartments, Related to Figure 3 (A) γδ IEL composition in adult WT versus *Btnl1*^*indel/indel*^ mice (n = *3*) (B) Mesenteric Lymph Node (MLN) and (C) Splenic immune compartments of WT, *Btnl1+/−* and *Btnl1−/−* analyzed by flow cytometry (n = *7–9*). (D) TCRVγ chain usage in MLN and splenic lymphocytes harvested from WT, *Btnl1+/−* and *Btnl1−/−* mice assessed by flow cytometry (n = *8*). (E) Vγ7^+^ and Vγ7^+^GL2^+^ thymocytes from WT or *Btnl1+/−* and *Btnl1−/−* mice assayed by flow cytometry to enumerate total cell counts (*left*) and cell surface phenotype (*right*) at three time points. Panels (A-D) are representative of 1 experiment. Panel E-*left* presents data pooled from ≥ 2 independent experiments. All error bars represent mean ± SD. See Table S3.

The four *Btnl1*^−/−^ strains each displayed major, highly selective losses of Vγ7^+^ IELs, assessed by flow cytometry or confocal microscopy (Figures 3A, 3B, and S3A). Vγ7^+^ IEL numbers were depleted by ∼90%, with Vγ7GL2^+^ cells almost ablated. Because Vγ7^−^ IEL numbers barely increased, the percentage representation of Vγ7^+^ cells among γδ IELs was reduced only by ∼3-fold relative to wild-type (WT) mice, but this was set against a background of dramatically reduced γδ IEL numbers (Figures 3A and S3A). By contrast, TCRβ^+^CD8αα^+^ IEL numbers increased significantly in *Btnl1*^−/−^ mice (Figures 3A and 3B).

The specificity of *Btnl1* for Vγ7^+^ IELs was emphasized by comprehensive immune phenotyping of *Btnl1*^−/−^, WT, and *Btnl1*^*+/*−^ mice that showed comparable splenic or MLN immune cell subsets (including γδ cell repertoires) and comparable representation and phenotypes of Vγ7^+^ thymocytes from day 4 to week 8 (Figures S3B–S3E). Consistent with its expression pattern, *Btnl1* acted extrathymically; thus, *Btnl1* deficiency crossed onto NU/NU mice reduced the average number of Vγ7^+^ IELs by ∼90%, with almost total loss of Vγ7GL2^+^ IELs (Figure 3C). Since NU/NU mice lack most αβ T cells, this result excludes the formal albeit unlikely possibility that Vγ7^+^ IEL losses in euthymic *Btnl1*^−/−^ mice indirectly reflected expanded TCR αβ IELs. The specificity of Vγ7^+^ IELs for *Btnl1* was emphasized by the fact that *Btnl4*^−/−^ mice displayed no overt defects in any major IEL subset (Figure 3D).

### *Btnl1* Selects Vγ7^+^ IEL In *Trans*

**Figure 4:**
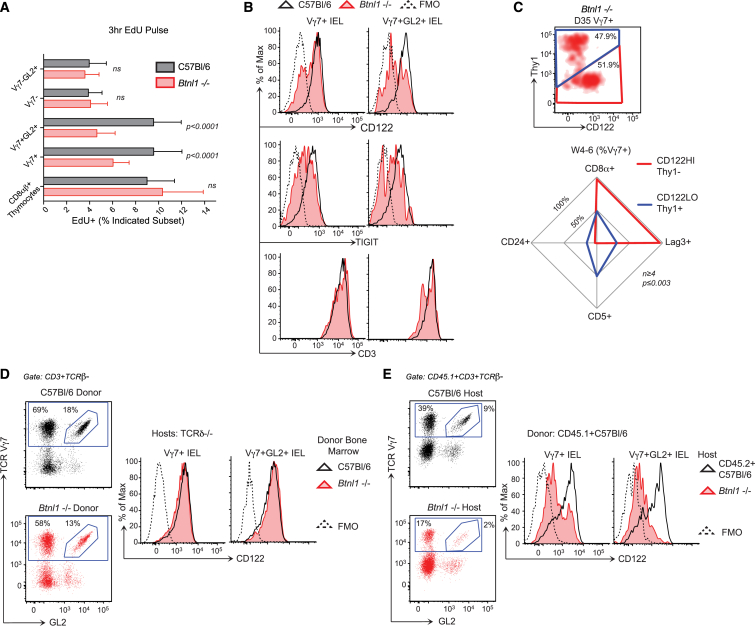
*Btnl1* Drives Selective Expansion and Maturation of Gut IEL (A) 3 hr EdU incorporation in vivo in γδ IEL subsets and thymocytes from week 4 WT versus *Btnl1*^−/−^ mice (n ≥ 9). (B) Surface phenotypes of Vγ7^+^ and Vγ7GL2^+^ IELs from week 3–5 WT and *Btnl1*^−/−^ mice (n ≥ 8). (C) Top: Thy1 and CD122 expression by Vγ7^+^ IEL from day 35 *Btnl1*^−/−^ mice (n = 8). Bottom: surface phenotypes of CD122^HI^Thy1^−^ and CD122^LO^Thy1^+^ Vγ7^+^ IELs from week 4–6 *Btnl1*^−/−^ mice (n ≥ 4). W, week. (D and E) IEL reconstitution and CD122 profiles in (D) irradiated TCRδ^−/−^ mice 9–10 weeks post-BM transfers from indicated donors (n ≥ 7) and (E) irradiated CD45.2^+^ WT or *Btnl1*^−/−^ mice 4–5 weeks post-BM transfers from CD45.1^+^ C57Bl/6 mice (n = 7). Data are representative of two (D and E) or three or more (C, top) independent experiments. Panels (A) and (C) (bottom) present data pooled from three or more experiments. All error bars represent mean ± SD. See also Figure S4.

**Figure S4:**
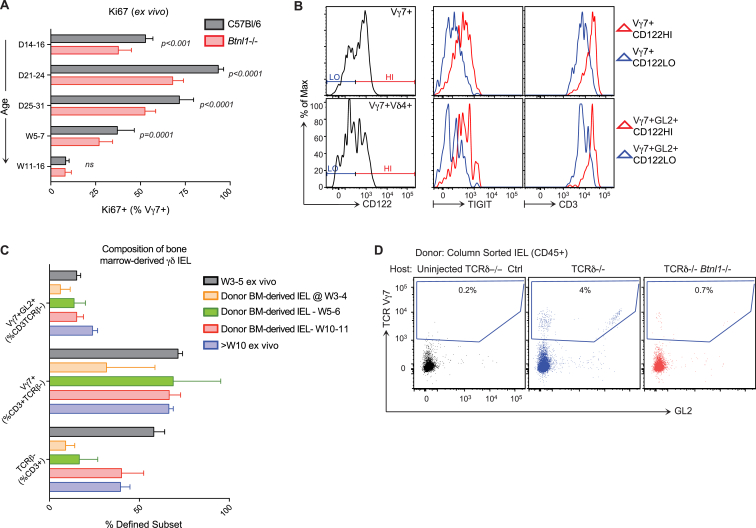
Impact of *Btnl1* on Intestinal Engraftment, Expansion, and Retention of CD122^HI^ Vγ7^+^ IELs, Related to Figure 4 (A) Ki67^+^ expression in Vγ7^+^ IEL isolated from WT versus *Btnl1*−/− mice (n = *4-27*). (B) Cell surface phenotype of *Btnl1*−/− Vγ7^+^CD122^HI^ versus Vγ7^+^CD122^LO^ and Vγ7^+^GL2^+^CD122^HI^ versus Vγ7^+^GL2^+^CD122^LO^ IEL displayed in Figure 4B (n ≥ 8). (C) Irradiated TCRδ KO mice reconstituted with WT bone marrow (BM) were analyzed for γδ IEL composition at the indicated time-points after BM transfer (n ≥ *3*). (D) IEL isolated from WT W4-5 mice were column-purified using CD45 microbeads and adoptively transferred intravenously into W6 TCRδ−/− or TCRδ−/−*Btnl1*−/− hosts. γδ T cell composition was assayed 2-3 weeks later by flow cytometry (n ≥ 5). Data are representative of 1 (C), 2 (D) or ≥ 3 (B) independent experiments. Bar graph displays mean ± SD. All error bars represent mean ± SD.

To determine how and when *Btnl1* impacts IELs, we examined residual Vγ7^+^ and Vγ7GL2^+^ IELs in *Btnl1*^−/−^ mice. Relative to those in WT mice, significantly fewer Vγ7^+^ IELs incorporated EdU at D28 [p < 0.0001] or expressed Ki67, and this did not change until W7 when most Vγ7^+^ IELs in WT mice moved out of cycling (Figures 4A and S4A). Thus, there was no selective expansion of Vγ7^+^ IELs in *Btnl1*^−/−^ mice. Similarly, although their TCR levels were high, many residual Vγ7^+^ and Vγ7GL2^+^ IELs in week 3–5 *Btnl1*^−/−^ mice were CD122^lo^, Thy1^+^, TIGIT^−^, Lag3^−^, CD8αα^−^, CD5^+^, CD24^+^, thereby phenocopying immature Vγ7^+^ IELs of day 14–17 WT mice (Figures 4B, 4C, and S4B).

To demonstrate that *Btnl1* exerts its selective impact on Vγ7^+^ T cells in *trans*, we established conditions in which the Vγ7^+^ and Vγ7GL2^+^ IEL compartments of week 3–5 WT mice were reconstituted within ∼5 weeks of donor bone marrow (BM) transfer to irradiated 8- to 10-week, γδ T cell-deficient TCRδ^−/−^ mice (Figure S4C). BM from either WT or *Btnl1*^−/−^ mice proved equally effective at IEL reconstitution (Figure 4D). Not surprisingly, WT BM reconstitution of Vγ7^+^ IELs in irradiated, congenic T cell-sufficient CD45.2^+^ WT recipients was less effective than it was in TCRδ^−/−^ hosts (compare plots, top left in Figures 4D and 4E), but nonetheless, reconstitution of *Btnl1*^−/−^ hosts was much less effective, and the few Vγ7^+^ and Vγ7GL2^+^ IELs that developed in *Btnl1*^−/−^ recipients phenocopied residual Vγ7^+^ cells in *Btnl1*^−/−^ mice and immature IELs in day 14–17 WT mice (Figure 4E). Complementing these findings, purified IELs from 4-week-old mice could reconstitute Vγ7^+^ IELs in recipient TCRδ^−/−^ mice, albeit very inefficiently, and this too was greatly impaired in *Btnl1*^−/−^TCRδ^−/−^ hosts (Figure S4D). Thus, *Btnl1* acts in non-hematopoietic cells to support the selective expansion and maturation of Vγ7^+^ IEL.

**Figure S5:**
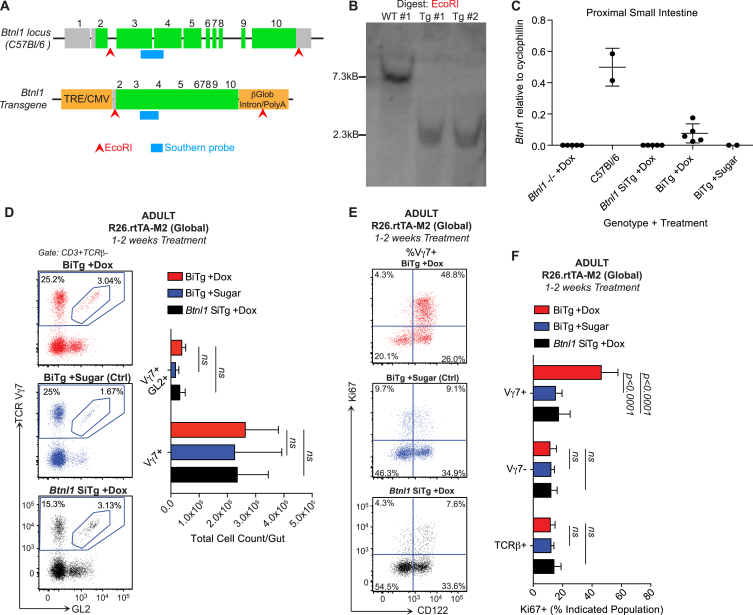
Inducible *Btnl1* Transgene Expression and Its Impact in Adult Mice, Related to Figure 5 (A) Schematic representation of the WT *Btnl1* locus (top) and TRE-*Btnl1* transgene construct (bottom). Grey: unstranslated region; green: translated region; orange: upstream-tetracycline response element/CMV promoter and downstream-β-globulin/polyA. (B) Southern blot to detect transgene insertion. Genomic DNA was digested with EcoR1 as indicated (arrowheads) and a probe (blue bar) targeting the indicated region (Exon3/4 boundary in ORF) was generated to detect the WT and targeted allele (n = *2*). C-F) W7-13 (ADULT) mice of indicated genotypes on a *Btnl1−/−* background were administered doxycycline water (1mg/ml Dox, 2% suchrose) or ctrl water (2% suchrose) for 1-2 weeks. (C) Gene expression by qRT.PCR in proximal small intestine of adult mice following the indicated treatment. (D) γδIEL composition (*left*) and absolute cell counts (*right*) assessed by flow cytometry in adult mice following the indicated treatment (*sugar, n = 3-5; rest, n = 4-10*). (E) Ki67 and cell surface CD122 expression in Vγ7^+^ IEL from adult mice following the indicated treatment. (F) Ki67 expression in Vγ7^+^ versus Vγ7^-^ and TCRβ^+^ IEL from adult mice following the indicated treatment (n = *5-9*). Data are representative of 2 (B), or 3 independent experiments. Some panels include results pooled from 2 (C) or ≥ 3 (D,F) independent experiments. All error bars represent mean ± SD.

To attempt to restore IEL selection, we rendered *Btnl1*^−/−^ mice transgenic for *Btnl1* expressed from a doxycycline (Dox)-inducible promoter (Figures S5A and S5B) and crossed them onto *Btnl1*^−/−^ mice, into which was introduced a Dox-responsive *trans*-activator (rtTA) regulated by the ubiquitously expressed *Rosa26* promoter. As reported, adding low-dose Dox to drinking water had little overt impact on the gut over a several-week period (Roth et al., 2009). Therefore, this system offered a means to globally induce *Btnl1* de novo in *Btnl1*^−/−^ bitransgenic (BiTg) mice that inherited both rtTA and the inducible *Btnl1* transgene. Conversely, BiTg mice administered sugar water and Dox-treated single-transgenic (SiTg) *Btnl1*^−/−^ mice (that inherited only the *Btnl1* transgene) served as controls. *Btnl1* induction in mice of appropriate genotypes was validated by qPCR (Figure S5C).

When several W11 *Btnl1*^−/−^ BiTg mice were treated in this way, the representation of Vγ7^+^ and Vγ7^+^GL2^+^ IELs was unchanged relative to littermate controls, but the percentage of Vγ7^+^ IELs that were CD122^hi^ increased greatly, and most expressed Ki67. This was not true for Vγ7^−^ or TCRβ^+^ IELs (Figures S5D–S5F). Thus, *Btnl1* induction de novo in adult mice partially phenocopied Vγ7^+^ IEL-specific maturation in early life but did not reconstitute Vγ7^+^ IEL numbers. Global *Btnl1* induction also did not drive ectopic Vγ7^+^ cell maturation in any other tissues (unpublished data).

### A Temporal Window for Epithelial *Btnl* Activity

**Figure 5:**
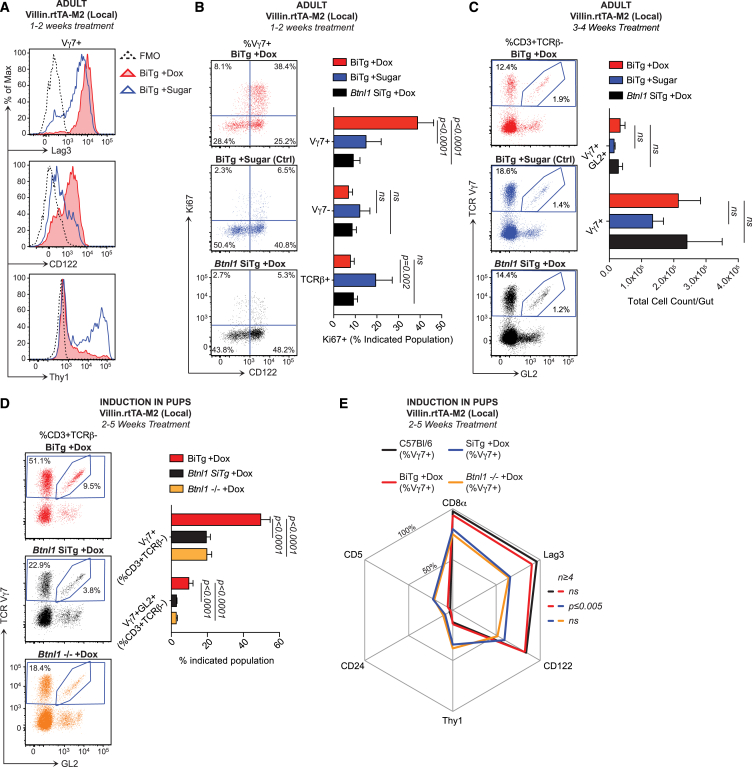
Villin-Specific *Btnl1* Induction Rescues Vγ7^+^ IEL In Vivo (A–D) Week 7–13 (adult) or day 7–21 (pups) mice of indicated genotypes on a *Btnl1*^−/−^ background were administered Dox (1 mg/ml, 2% sucrose) or control water (2% sucrose) for times indicated, and IELs were analyzed by flow cytometry. n ≥ 5 (A and B); n ≥ 6 (C and D). (E) Comparative cell-surface phenotypes of Vγ7^+^ IELs from week 4–5 WT mice and animals indicated (n = 4–8). (A) is representative of two or more independent experiments; (B)–(E) present data pooled from three or more experiments. Statistical significance in (C) was determined using the Holm-Sidak method. All error bars represent mean ± SD. See also Figure S5.

In further experiments, we restricted *Btnl1* induction to mature enterocytes by generating *Btnl1*^−/−^ mice transgenic for rtTA expressed from the villin promoter. Within 1–2 weeks of Dox-treatment of several W11 BiTg *Btnl1*^−/−^ mice, most Vγ7^+^ IELs had become Lag3^hi^, Thy1^−^, and CD122^hi^, of which the majority were also Ki67^+^ (Figures 5A and 5B). Again, this phenotypic transition was Vγ7^+^ IEL specific, albeit there were sometimes *Btnl1*-independent increases in Ki67^+^ TCRβ^+^ IELs in sugar-water-treated mice. (Figure 5B). Once more, the numbers and representation of Vγ7^+^ IELs were unchanged, even in mice retained on Dox for 3–4 weeks (Figure 5C). This establishes that *Btnl1* can effect phenotypic conversion of immature Vγ7^+^ IELs rather than merely promote a selective outgrowth of mature Vγ7^+^ cells.

However, there was significantly increased representation of Vγ7^+^ and Vγ7GL2^+^ IEL when *Btnl1* expression was induced in *Btnl1*^−/−^ mice in early-life by commencing Dox-treatment of nursing females at D7 or of weanlings at D21 and then maintaining treatment for 2-5 weeks (Figure 5D). Moreover, the expanded Vγ7^+^ cells phenocopied IEL of W4-5 WT mice, and were significantly different from the IEL of Dox-treated SiTg mice and *Btnl1*^*−/−*^ mice (Figure 5E). Thus, acute expression of *Btnl1* purely in the gut epithelium induces the selective phenotypic maturation and expansion of Vγ7^+^ IEL, but these effects are separable, with selective expansion mostly confined to a developmental window within the first five weeks of life.

### Epithelial Btnl1 and Btnl6 Regulate Vγ7 Cells

**Figure S6:**
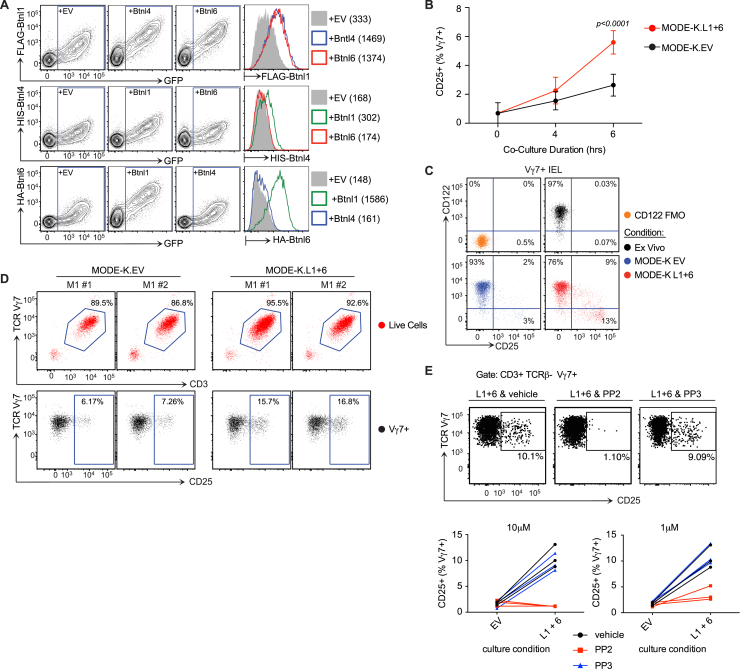
Co-expression of Btnl1 and Btnl6 and Their Impact on Vγ7^+^ IELs, Related to Figure 6 (A) Cell surface expression of FLAG-Btnl1, HIS-Btnl4 or HA-Btnl6 co-transfected in MODE-K cells. Histogram overlays show the expression of each BTNL after gating on GFP+ cells (numbers in brackets indicate geometric mean fluorescence intensity, gMFI). (B) Primary small intestinal IEL cultured for the indicated times with MODE-K cells transduced with constructs expressing an empty vector (EV) versus *Btnl1*+*Btnl6* (L1+6) (n = *7*). (C) Representative plots of cell surface CD122 and CD25 expression on Vγ7+ cells after the indicated overnight culture conditions (n = *21*). (D) Cell surface CD25 expression in positively FACS-sorted Vγ7+ IEL after overnight co-culture with MODE-K cells expressing EV versus L1+6 (n = *4*). (E) Cell surface CD25 expression in primary Vγ7^+^ IEL after overnight co-culture with the indicated MODE-K transductants in the presence PP2, PP3 or vehicle. Data are representative of representative of 2 (A,D), or > 5 (C) independent experiments. Some panels (B,E) present data pooled from 2 independent experiments. All error bars represent mean ± SD.

Given that acute *Btnl1* expression drives the selective maturation of Vγ7^+^ IELs in vivo, we tested whether *Btnl1* might show specificity for Vγ7^+^ IEL ex vivo. Since primary intestinal epithelial cells reportedly harbor Btnl1 in a complex with Btnl6 (Lebrero-Fernández et al., 2016), we sought evidence for heterotypic interactions of Btnl proteins. Indeed, cell surface expression of Btnl1 on *Btnl1-*transfected MODE-K cells (an established intestinal epithelial cell line in which endogenous *Btnl* genes are negligibly expressed) was greatly enhanced by co-transfection with *Btnl4* or *Btnl6* (Figure S6A). Likewise, surface Btnl6 expression was greatly enhanced by Btnl1, but there was no evidence for collaboration between Btnl6 and Btnl4 (Figure S6A). Given the specificity of Btnl6 for Btnl1, and given that *Btnl4*^−/−^ mice showed no IEL phenotype, we focused on Btnl1 and Btnl6.

**Figure 6:**
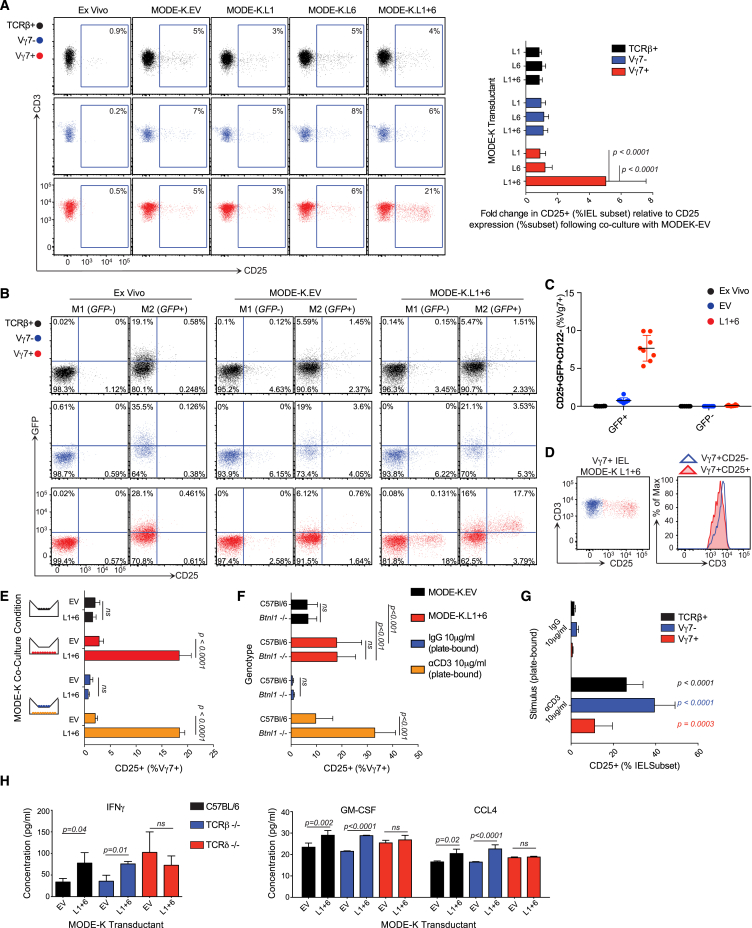
Gut Vγ7^+^ IELs Respond to Btnl Proteins (A) Left: surface CD25 expression of designated IEL subsets after 12-hr co-culture of total IELs with MODE-K cells transduced with empty vector (EV), *Btnl1* (L1), *Btnl6* (L6), or *Btnl1* plus *Btnl6* (L1+6). Right: fold increase in CD25^+^ cells as percentage of the IEL subset relative to co-culture with MODE-K.EV (n = 21). (B) GFP and CD25 expression by designated IELs from Nur77.gfp mice ex vivo or after 12-hr co-culture of total IELs with designated cells (n = 8). (C) Percentage of Vγ7^+^ IELs from Nur77.gfp mice that were CD25^+^GFP^+^CD122^−^ directly ex vivo or after 12-hr co-culture of total IELs with designated cells. (D) CD3 expression in Vγ7^+^CD25^+^ or Vγ7^+^CD25^−^ IELs after 12-hr co-culture of total IELs with MODE-K.L1+6 (n = 21). (E) Surface CD25 expression as percentage of Vγ7^+^ IELs after indicated transwell co-culture conditions (n = 3). (F) Surface CD25 expression on Vγ7^+^ IELs from WT and *Btnl1*^−/−^ mice after indicated culture conditions (n = 7). (G) Surface CD25 expression by designated IEL subsets after incubation with anti-CD3 or control immunoglobulin (Ig) (n ≥ 12). (H) Cytokine concentrations assessed by luminex in supernatants after 48 hr of co-cultures indicated (n = 3). Data are representative of one (H), two (B) or more than three (A, D, and E) independent experiments. Some panels present data pooled from two (C) or more than three (A, F, and G) independent experiments. All error bars represent mean ± SD. See also Figure S6.

MODE-K cells stably transduced with *Btnl1* (L1), *Btnl6* (L6), *Btnl1* plus *Btnl6* (L1+6), or empty vector (EV) were co-cultured with freshly explanted IELs that were then assayed for CD25 (IL-2Rα chain) upregulation, which is among the most robust readouts of TCR stimulation for systemic T cells (Depper et al., 1984, Kim and Leonard, 2002). Whereas mixed TCRαβ^+^ and TCRγδ^+^ IELs showed minor CD25 upregulation upon co-culture with EV, L1, or L6 cells, IELs exposed to L1+6 cells showed highly significant CD25 upregulation, wholly attributable to ∼20% of Vγ7^+^ cells (both Vγ7GL2^+^ and Vγ7GL2^−^ cells) (Figure 6A). Significant CD25 upregulation was first evident within 4–6 hr, as is true for systemic TCR stimulation (Depper et al., 1984, Kim and Leonard, 2002) (Figure S6B).

When L1+6 cells were co-cultured with primary IELs from Nur77.*gfp* mice in which GFP is upregulated by nuclear factor of activated T cells (NFAT) activation downstream of TCR signaling (Moran et al., 2011), essentially all IELs that upregulated CD25 were GFP^+^ (Figure 6B). This phenotype was not shown by Vγ7^−^ or TCRαβ^+^ IELs in the same co-cultures (Figure 6B). IEL upregulating CD25 also downregulated CD122, an additional symptom of systemic TCR stimulation (Yu and Malek, 2001) (Figure S6C). Thus, CD25^+^GFP^+^CD122^−^ cells arose uniquely among Vγ7^+^ IEL co-cultured with L1+6 cells (Figure 6C). Moreover, CD25 upregulation was accompanied by slight but significant TCR downregulation, another rapid response to TCR stimulation (San Jose et al., 2000) (Figure 6D). Btnl1+Btnl6 acted directly on Vγ7^+^ IEL, since CD25 was upregulated on cells that had been purified by flow cytometry prior to L1+6 cell co-culture (Figure S6D). (Note that background CD25 expression was increased by TCR-dependent sorting, but this did not obscure the result.)

When IEL were separated from L1+6 cells by transwells, CD25 upregulation was abrogated and could not be secondarily *trans-*activated by IELs that were in contact with L1+6 cells, e.g., via secreted cytokines (Figure 6E; compare blue with yellow bars in bottom panel). CD25 upregulation by IELs in contact with L1+6 cells showed dose-dependent inhibition by PP2, which inhibits signaling by src-family kinases, such as *Lck* and *Fyn*, but was not inhibited by PP3, an established control for PP2 specificity (Figure S6E).

Just as residual Vγ7^+^ IEL in *Btnl1*^−/−^ mice responded to acute transgenic *Btnl1* induction in vivo, they were comparable to WT Vγ7^+^ IELs in responding to Btnl1 plus Btnl6 ex vivo (Figure 6F). Interestingly in the same experiments, WT Vγ7^+^ IELs showed relatively poor responses to anti-CD3, phenocopying the attenuated responsiveness imposed on Vγ5^+^ DETC progenitors by *Skint1* (Wencker et al., 2014), whereas Vγ7^+^ IELs from *Btnl1*^−/−^ mice, and TCRαβ^+^ and Vγ7^−^ IELs from WT mice, none of which subsets had experienced prior *Btnl1* selection in vivo, all showed strong responses to anti-CD3 (Figures 6F and 6G).

Finally, the supernatants of IEL co-cultures with L1+6 cells showed small but significant increases in interferon-γ (IFN-γ), CCL4, and granulocyte macrophage colony-stimulating factor (GM-CSF) among 36 cytokines tested (Figure 6H). These are typical IEL effectors, and although it was technically challenging to attribute production to Vγ7^+^ IELs, such increases were not seen in supernatants of L1+6 cells cultured with IELs from TCRδ^−/−^ mice. (Note the higher background cytokine expression in TCRδ^−/−^ mice may reflect spontaneous inflammation often associated with γδ deficiency; Hayday and Tigelaar, 2003.) In sum, a range of metrics attested to a highly specific and direct interaction ex vivo of Vγ7^+^ IELs with Btnl1 and Btnl6 co-expressed on gut epithelial cells.

### Signature Human Intestinal γδ T Cells

**Figure 7:**
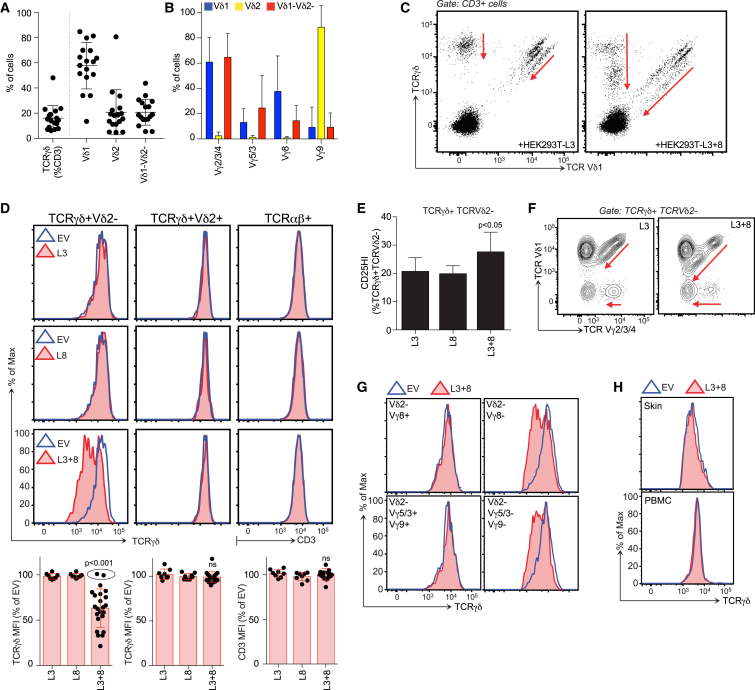
Regulation of Human Gut Vγ4^+^ Cells by BTNL3 and BTNL8 (A and B) Vδ (A, n = 17) and Vγ (B, n = 6–10) expression by human gut γδ cells. (C) Surface TCRγδ/Vδ1 expression on human gut lymphocytes after 12-hr co-culture with BTNL3 (L3) or BTNL3 plus BTNL8 (L3+8)-transduced HEK293T cells. Red arrows denote shifts in TCR staining. (D) Top: TCRγδ/CD3 expression on designated human gut T cells after 12-hr co-culture with denoted HEK293T transductants. Bottom: mean fluorescence intensities (MFIs) calculated relative to co-culture with HEK293T.EV (n ≥ 22). For two donors, MFIs for Vδ2^−^ cells remained unchanged (dots within the ellipse). (E) Percentage of CD25^HI^ cells among TCRγδ^+^TCRVδ2^−^ T cells after co-culture with denoted cells (n = 5). Statistical analysis was performed by paired t test. (F) Surface Vγ2/3/4 and Vδ1 expression on Vδ2^−^ γδ T cells after co-culture with cells denoted. (G) TCRγδ expression on indicated subsets after co-culture with denoted cells. (H) TCR**γδ** expression on **γδ** cells from peripheral blood mononuclear cells (PBMCs) or skin after co-culture with denoted cells. All error bars represent mean ± SD. See also Figure S7 and Table S1.

Because of limited tissue access, human gut T cells are understudied. Nonetheless, there are gut-associated γδ cells whose TCR usage differs markedly from Vγ9^+^Vδ2^+^ cells that dominate the peripheral blood (Landau et al., 1995). To better characterize such cells, we submitted biopsy specimens from healthy ascending colon to a modified version of a protocol used to isolate human skin T cells (Clark et al., 2006). For 16 of 17 donors, the γδ cells were enriched in Vδ1^+^ cells, although Vδ1^−^Vδ2^−^ cells were also present; hence, the term “Vδ2^−^” is used to distinguish tissue-associated γδ cells from Vδ2^+^ cells that could also be recovered from most gut samples, albeit in highly variable numbers (Figure 7A).

**Figure S7:**
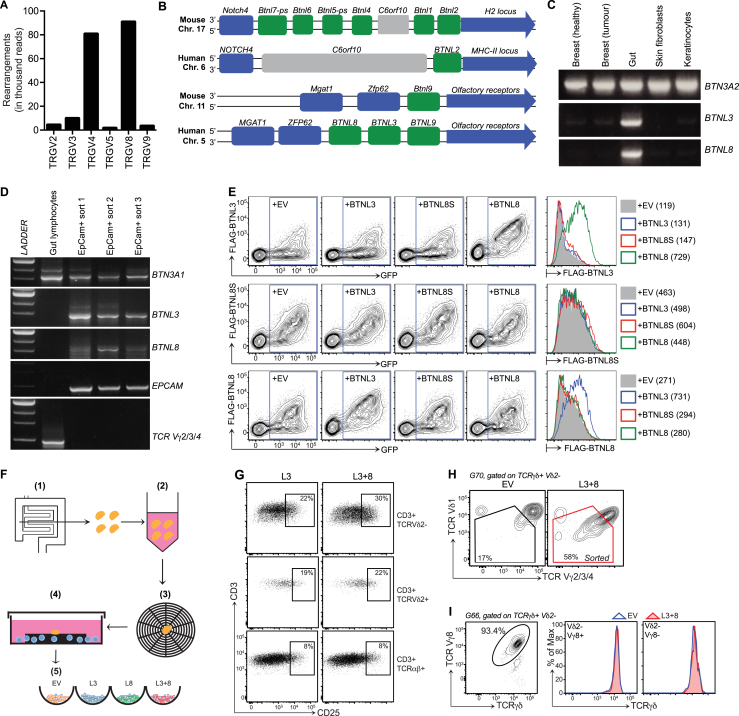
Human Intestinal γδ Cells and the Selective Impact on Them of BTNL3 and BTNL8 Co-expression, Related to Figure 7 (A) FACS-sorted γδ T cells harvested from human intestinal tissue were analyzed by deep sequencing for TCR Vγ chain usage. (B) Schematic illustrating the murine and human Btnl2/BTNL2 and Btnl9/BTNL9 loci, adapted from the NCBI gene viewer. (C) Conventional RT-PCR analysis of *BTN3A2*, *BTNL3* and *BTNL8* expression in the indicated tissues. (D) Conventional RT-PCR analysis of *BTN3A1*, *BTNL3*, *BTNL8*, *EPCAM* and TCR Vγ2/3/4 expression in the indicated samples. (E) Cell surface expression of FLAG-BTNL3, FLAG-BTNL8S or FLAG-BTNL8 co-transfected in HEK293 cells with the indicated constructs. Histogram overlays show the expression of each BTNL after gating on GFP+ cells (numbers in brackets indicate geometric mean fluorescence intensity, gMFI). (F) Schematic illustrating the method of human intestinal tissue-resident lymphocytes isolation and co-culture with HEK293 transductants. (1) Endoscopic biopsies recovered from ascending colon of healthy donors. (2) Washed in complete media supplemented with antibiotic. (3) 1 biopsy applied to each matrix. (4) Culture for 5-7 days in complete medium supplemented with antibiotics, IL-2 and IL-15. (5) Co-culture with HEK293 cell lines transduced with EV, L3, L8 or L3+8. (G) Cell surface CD25 expression on indicated subsets of human gut-derived lymphocytes after co-culture with EV versus L3+8-transduced HEK293 cells. (H) Gating parameters for sorting of Btnl3+8-responsive human gut-derived lymphocytes. (I) TCRVγ chain usage (*left*) and cell surface TCRγδ expression (*right*) in gut-derived γδ T cells (isolated from a donor unresponsive to BTNL3+8) after co-culture with EV versus L3+8-transduced HEK293 cells.

Of six functional human Vγ chain genes (Vγ2, 3, 4, 5, 8, and 9) (Arden et al., 1995), Vγ4 was reported to be the signature chain of intestinal Vδ2^−^ cells (Landau et al., 1995). Indeed, for up to ten donors examined, most intestinal Vδ2^−^ cells reacted with a Vγ2/3/4-specific antibody, but not a Vγ5/3-specific antibody (blue and red bars, Figure 7B; Table S1A), and TCR deep sequencing showed that Vγ4 sequences far outnumbered Vγ2 sequences (Figure S7A). Thus, despite individual variation, most gut γδ T cell compartments included a substantial Vγ4^+^Vδ2^−^ subset, while some donors also displayed relatively high representation of Vγ8^+^Vδ1^+^ cells (Figure 7B; Table S1A).

### BTNL3 and BTNL8 Regulate Human Vγ4^+^ Cells

There is no human equivalent of the *Btnl2-*proximal amplicon on mouse chromosome 17 that encodes *Btnl1*, *Btnl4*, and Btnl6 (Abeler-Dörner et al., 2012, Afrache et al., 2012). However, adjacent to human *BTNL9* is an amplicon that encodes *BTNL3* and *BTNL8* whose expression is highly enriched in gut, particularly EpCAM^+^ epithelial cells (Figures S7B–S7D). Interestingly, akin to the behavior of Btnl1 and Btnl6 described above, neither BTNL3 nor BTNL8 protein was efficiently expressed on cells transfected with their respective genes, unless both were co-expressed (Figure S7E, green line, top right histogram; blue line, bottom right histogram). Conversely, BTNL8S (a splice variant of BTNL8) failed to rescue surface BTNL3 expression (Figure S7E, red line, top right histogram).

Whereas we could not test for a developmental dependence of human gut γδ cells on BTNL3 and BTNL8, we could assess whether BTNL3 and BTNL8 phenocopied Btnl1 and Btnl6 by specifically activating signature gut γδ cells in a TCR-dependent fashion. Thus, we established short-term co-cultures of primary-gut-derived lymphocytes with HEK293T cells transduced with *BTNL3* (L3), *BTNL8* (L8), *BTNL3* and *BTNL8* (L3+8), or EV (Figure S7F). For the representative donor shown, some of the discrete subsets of Vδ1^−^ and Vδ1^+^ γδ cells that were apparent in T cell co-cultures with control (EV, L3, and L8) cells showed marked TCR downregulation when co-cultured with L3+8 cells (red arrows Figure 7C). Emphasizing specificity, TCR downregulation occurred in response to L3+8 cells in 21 of 23 donors but was never seen in co-cultures with L3 or L8 cells, and was never shown by intestinal Vδ2^+^ or TCRαβ^+^ cells, even in the same cultures as responding Vδ2^−^ cells (Figure 7D). Although higher baseline CD25 expression reduced the sensitivity of this assay for human versus mouse gut T cell activation ex vivo, L3+8 cells induced significant CD25 upregulation vis-a-vis gut T cells co-cultured with control cells (Figure 7E), and CD25 upregulation was most evident on cells with downregulated TCRs (Figure S7G).

Not all Vδ2^−^ cells responded to L3+8 (Figure 7C). Thus, we considered that TCRγ chains might determine BTNL responsiveness, as is true in mice. Indeed, human Vδ2^−^ populations that downregulated TCRs in co-cultures with L3+8 cells were detected by the Vγ2/3/4-specific antibody, but not by antibodies to Vγ8, Vγ5/3, or Vγ9 (Figures 7F and 7G). Moreover, productively rearranged Vγ4 genes were prevalent when L3+8-responsive cells with downregulated TCRs were flow cytometry sorted from one donor (Figure S7H) and their Vγ chains amplified without bias and sequenced (red notation, Table S1B). By contrast, TCRγ transcripts from skin-derived TCRγδ^+^ cells (G234SK01) were biased toward Vγ3 (purple notation Table S1B). Interestingly, of two donors showing no substantial response to BTNL3+8, one proved a posteriori to have an atypical intestinal γδ T cell repertoire dominated by Vγ8^+^ cells (Figure S7I). Likewise, L3+8 cells induced no significant TCR down-modulation by primary γδ^+^ T cells from skin or blood among which Vγ4^+^ cells are rare (Figure 7H). Thus, epithelial *BTNL* genes regulate human-tissue-resident γδ T cells in an organ-specific, TCRγ-chain-specific manner.

## Discussion

This study shows that the unique composition of the murine intestinal intraepithelial T cell compartment arises from a selective maturation and expansion of Vγ7^+^ T cells driven by *Btnl1*, and most likely *Btnl6*, expressed by differentiated enterocytes. Likewise, *BTNL3* and *BTNL8* co-expressed by human intestinal epithelial cells selectively regulate gut Vγ4^+^ T cells. Given that *Skint1* expressed by thymic epithelial cells and keratinocytes selectively regulates intra-epidermal Vγ5^+^ T cells, tissue-specific *Btnl* genes may offer a generalizable means by which epithelia shape and regulate local γδ T cell compartments. This may reflect an even broader utilization of *BTNL*/*BTN* genes in γδ biology, since BTN3A1 is critical to human peripheral blood γδ cell activation (Harly et al., 2012, Palakodeti et al., 2012, Vavassori et al., 2013, Wang et al., 2013).

*Btnl1* and *Skint1* effect many of the same changes in Vγ7^+^ and Vγ5^+^ IEL progenitors, respectively. These include upregulation of the receptor for IL15, a growth factor expressed by epithelial cells essential for γδ IEL maintenance (De Creus et al., 2002, Lai et al., 2008, Lodolce et al., 1998); suppression of *sox13*, *rorgc*, and *bcl11b* that are associated with γδ cells producing IL-17, which is not a property of IELs (Jensen et al., 2008, Turchinovich and Hayday, 2011); and attenuation of TCR responsiveness, consistent with IELs adopting an innate-like, rapidly responsive, tissue surveillance role (Wencker et al., 2014).

Being members of the B7 superfamily, *Btnl*/*BTNL*/*Skint* gene products may act as co-stimulators for IEL receptors yet to be identified. In this case, they will be the first co-stimulators specific for cells with particular TCRs; e.g., Vγ7^+^, but not Vγ7^−^, IELs from mouse gut; and Vγ4^+^, but not Vγ8^+^, T cells from human gut. Alternatively, their exquisite specificities may reflect interactions of Btnl/BTNL/Skint with cognate TCRs, possibly via unique Vγ-CDR1/2 regions. Likewise, human BTN3A1 appears to mediate its effects via the Vγ9Vδ2 TCR, although there are no clear direct binding data. This may reflect a highly complex interaction that includes critical co-factors. Of note, BTNL3, BTNL8, Btnl1, and Btnl6 each contain B30.2 domains related to the phosphoantigen-binding domain of BTN3A1, raising the possibility that low-molecular-weight metabolite(s) might have a broad role in γδ cell regulation by *Btnl*/*BTNL* genes (Adams et al., 2015). A requirement for co-factors might also explain the developmental time window during which Btnl1 could drive the maturation and expansion of Vγ7^+^ IELs.

Peptide-MHC complexes have different impacts on αβ T-lineage cells, including positive and negative selection of thymocytes, T-regulatory cell differentiation, and activation or anergy of mature peripheral T cells (Burkly et al., 1989, Fink and Bevan, 1978, Jenkins and Schwartz, 1987, Jordan et al., 2000). These outcomes are dictated by the state of the T cell and/or the biological context. In so far as parallels may be drawn, our study offers genetic evidence that *Btnl1* drives Vγ7^+^ IEL selection and cell biological evidence that Btnl1 and Btnl6 can promote weak activation of Vγ7^+^ IELs, which was likewise true for human BTNL3 plus BTNL8 interactions with mature gut γδ cells. These different contexts might explain a seeming paradox that CD122 is upregulated by *Btnl1*-mediated selection in vivo, in common with agonist selection of thymocytes (Hanke et al., 1994), and yet is downregulated by Btnl1 plus Btnl6-mediated cell activation ex vivo, in common with conventional T cell activation (Yu and Malek, 2001).

Weak activation of mature IEL may reflect the use by epithelia of site-specific *Btnl*/*BTNL* molecules to sustain cognate IELs, orienting them as to their correct anatomical location and to the status of the tissue. Consistent with this, Skint1 constitutively engages Vγ5^+^ DETCs (our unpublished data); Btnl1 can directly appose IEL at steady state (Bas et al., 2011), and the same may be true for BTNL3 and BTNL8 in humans, given RNAscope evidence that human colonic γδ cells are largely intraepithelial (our unpublished data). Such findings emphasize intimate and specific relationships between resting epithelial cells and neighboring T cells that provide a basis for tissue surveillance.

Although γδ IELs make essential contributions to body surface integrity (Hayday, 2000, Komano et al., 1995, Vantourout and Hayday, 2013), our identification of Btnl/BTNL proteins as key IEL regulators offers to clarify the contexts in which those contributions are most important. For example, when during gut development, dysregulation, and/or repair might epithelial Btnl/BTNL proteins and/or co-factors communicate a need for IEL activation? Such insight can inform genome-wide association study (GWAS) data implicating *BTNL*/*BTN* polymorphisms in numerous immunopathologies (Prescott et al., 2015, Rhodes et al., 2016, Valentonyte et al., 2005) and may reveal why epithelia use organ-specific, as opposed to pan-epithelial, *Btnl* gene products to regulate local T cells. In fact, different γδ TCRs offer IELs a means by which to discriminate organ-specific epithelia that is not obviously available to innate lymphoid cells.

Our and others’ studies have by now implicated Btnl1, Btnl6, Skint1, BTNL3, BTNL8, BTN3A1, and BTN3A2 in γδ cell regulation. Nonetheless, they may be pleiotropic. For example, the long cytoplasmic tails of BTNL3, BTNL8, Btnl1, and Btnl6 may signal back to the cells that display them, consistent with our finding that *Btnl1* attenuates epithelial cell sensitivity to IEL-derived cytokines (Bas et al., 2011). Yet, other *BTNL*/*BTN* protein functions may not relate to γδ cell biology (Rhodes et al., 2016), consistent with which we show that Btnl4 is not critical for gut γδ T cell development. A similar diversity of immunological functions may describe avian *Btnl*-like B-G genes, a subset of which may shape and/or regulate large γδ cell compartments in birds (Kaufman et al., 1999).

A clear exception to the many parallels of *Btnl1*-mediated IEL selection and *Skint1*-mediated DETC selection is *Skint1* expression by thymic epithelial cells (Boyden et al., 2008). Conversely, the restriction of *Btnl1* to the intestine, both naturally and in villin-driven BiTg mice, clearly establishes that the signature gut IEL compartment is shaped extrathymically, even if progenitors are thymus derived, thus resolving a long-standing controversy (Lefrançois, 1991, Poussier and Julius, 1994). This thymic independence may reflect a need to replenish gut IELs so as to maintain gut integrity post-thymic involution.

## STAR★Methods

### Key Resources Table

**Table:** 

REAGENT or RESOURCE	SOURCE	IDENTIFIER
**Antibodies**		

CD3 APC Cy7 (17A2)	BioLegend	Cat#:100222
CD3 PerCPCy5.5 (145-2C11)	BioLegend	Cat#:100328
TCRβ Brilliant Violet 421 (H57-597)	BioLegend	Cat#:109229
TCRβ APC (H57-597)	BioLegend	Cat#:109212
CD122 PE (TMβ1)	BioLegend	Cat#:123209
CD122 Brilliant Violet 421 (TMβ1)	BD	Cat#:562960
CD122 APC (TMβ1)	BioLegend	Cat#:123213
TIGIT PE (GIGD7)	eBiosciences	Cat#:12-9501-82
CD45RB APC Cy7 (C363-16A)	BioLegend	Cat#:C363-16A
Thy1.2 Brilliant Violet 510 (53-2.1)	BioLegend	Cat#:140319
Lag3 PerCP-efluor 710 (C9B7W)	eBioscience	Cat#:46-2231-80
CD5 PE (53-7.3)	BD PharMingen	Cat#:553023
CD24 FITC (M1/69)	eBiosciences	Cat#:11-0242-81
CD24 PECy7 (M1/69)	BD PharMingen	Cat#:560536
CD8α PECy7 (53-6.7)	BioLegend	Cat#:100722
TCR Vδ4 FITC (GL-2)	BD	Cat#:552143
TCR Vδ4 PE (GL-2)	BioLegend	Cat#:134905
CD8β PerCpCy5.5 (YTS156.7.7)	BioLegend	Cat#:126609
CD25 PerCpCy5.5 (PC61)	BioLegend	Cat#:102030
CD69 PECy7 (H1.2F3)	BioLegend	Cat#:104512
CCR9 PECy7 (CW-1.2)	BioLegend	Cat#:128711
CD44 PECy7 (IM7)	BioLegend	Cat#:103030
TCRVγ7 (F2.67)	Institut Pasteur, Paris, France, Pablo Pereira	N/A
TCRVγ1 APC (2.11)	BioLegend	Cat#:141107
TCRVγ4 APC (UC3-10A6)	BioLegend	Cat#:137708
TCRδ BV421 (GL3)	BioLegend	Cat#:118119
Ki67 FITC (B56/MOPC-21)	BD PharMingen	Cat#:556026
CD45 Qdot 605 (30-F11)	eBiosciences	Cat#:93-0451-42
CD5 Brilliant Violet 510 (53-7.3)	BD	Cat#:563069
TCRδ PeCy7 (GL3)	BioLegend	Cat#:118124
CD161/NK1.1 Brilliant Violet 650 (PK136)	BioLegend	Cat#:108735
CD4 Brilliant Violet 786 (GK1.5)	BD	Cat#:563331
CD8α AlexaFluor 700 (53-6.7)	BD	Cat#:557959
CD25 APC (PC61)	BD	Cat#:557192
GITR PE (DTA-1)	BD	Cat#:558119
CD44 FITC (IM7)	BD	Cat#:553133
CD62L PerCP-Cy5.5 (MEL-14)	BD	Cat#:560513
KLRG1 BV421 (2F1)	BD	Cat#:562897
CD11c BV786 (HL3)	BD	Cat#:563735
CD11b BV510 (M1/70)	BioLegend	Cat#:101245
F4/80 PerCPCy5.5 (BM8)	BioLegend	Cat#:123128
Ly6G APC (1A8)	BD	Cat#:560599
Ly6C AlexaFluor 700 (AL-21)	BD	Cat#:561237
CD103 PE (M290)	BD	Cat#:557495
CD317 Brilliant Violet 650 (927)	BioLegend	Cat#:127019
MHCII/IA/IE FITC (2G9)	BD	Cat#:553623
CD86 Pe-Cy7 (GL1)	BD	Cat#:560582
CD3 Brilliant Violet 421 (145-2C11)	BD	Cat#:562600
CD19 Brilliant Violet 421 (1D3)	BD	Cat#:562701
CD161/NK1.1 (lin) Brilliant Violet 421 (PK136)	BioLegend	Cat#:108735
IgG1 PE (A85-1)	BD	Cat#:550083
B220 (CD45R) AlexaFluor 700 (RA3-6B2)	BD	Cat#:557957
IgM Brilliant Violet 786 (R6-60.2)	BD	Cat#:564028
IgD PerCPCy5.5 (11-26c.2a)	Biolegend	Cat#:405710
GL-7 AlexaFluor 647 (GL7)	BD	Cat#:561529
CD95 PECy7 (Jo2)	BD	Cat#:557653
CD138 Brilliant Violet 650 (281-2)	BioLegend	Cat#:142517
CD21/35 FITC (7G6)	BD	Cat#:553818
CD23 Brilliant Violet 421 (B-ly6)	BD	Cat#:563929
DYKDDDDK-PE (Flag)	BioLegend	Cat#:627310
DYKDDDDK-APC (Flag)	BioLegend	Cat#:627308
HA-DyLight 650	Thermo Fisher	Cat#:26183-D650
6x-Histidine-PE	Abcam	Cat#:Ab72467
CD25 Brilliant Violet 421 (BC96)	Biolegend	Cat#:302630
CD25 PE (BC96)	Biolegend	Cat#:302606
CD3 Brilliant Violet 510 (OKT3)	Biolegend	Cat#:317332
CD3 BUV (UCHT 1)	BD Biosciences	Cat#:563546
EpCAM eFlour® 660 (1B7)	eBioscience	Cat#:50-9326
Streptavidin APC-Cy7	Biolegend	Cat#:405208
Streptavidin Brilliant Violet 421	Biolegend	Cat#:405225
TCRγδ PeCy7 (IMMU510)	Beckman Coulter	Cat#:41116015
Vγ9 PC5 (IMMU360)	Beckman Coulter	Cat#:41116015
Vγ9 PE (B3)	Biolegend	Cat#:331308
Vδ1 APC (REA173)	Miltenyi	Cat#:130-100-519
Vδ1 FITC (TS8.2)	Thermo Scientific	Cat#:TCR2730
Vδ2 PerCP (B6)	Biolegend	Cat#:331410
Vγ2/3/4 biotin (23D12)	D Kabelitz and D Wesch, University of Kiel	N/A
Vγ3/5 biotin (56.3)	D Kabelitz and D Wesch, University of Kiel	N/A
Vγ8 biotin (R4.5.1)	D Kabelitz and D Wesch, University of Kiel	N/A
Hamster IgG Isotype Control	Biolegend	Cat#:400933
LEAF-Purified anti-mouse CD3-ε	Biolegend	Cat#:100331

**Chemicals, Peptides, and Recombinant Proteins**		

BrdU	Sigma-Aldrich	Cat#:B5002
IL-2	Immunotools	Cat#:12340024
IL-15	Immunotools	Cat#:12340155
IL-3	R&D Systems	Cat#:403-mL
IL-4	R&D Systems	Cat#:404-mL
Amphotericin B	Thermo Scientific	Cat#:04195780D
Gentamicin	Sigma Aldrich	Cat#:G1272
Human recombinant IL-15	Biolegend	Cat#:570308
Human recombinant IL-2 (Proleukin)	Novartis Pharmaceuticals, Supplied by Guy’s Hospital pharmacy	N/A
Metronidazole	Baxter healthcare	Cat#:FE3400G

**Commercial Assays**		

Deep sequencing: Amp2Seq (illumina MiSeq)	Irepertoire	http://www.irepertoire.com/
Deep sequencing: immunoSEQ Platform	Adaptive biotechologies	http://www.adaptivebiotech.com/immunoseq
Zombie NIR™ Fixable Viability Kit	Biolegend	Cat#:423106
Live/Dead Fixable Blue Dear Cell Stain Kit	Thermo Fisher	Cat#:L23105
Click-iT EdU Alexa Fluor 647 Flow Cytometry Assay Kit	Invitrogen	Cat#:A10202
Foxp3 Staining Buffer Set	eBioscience	Cat#:00-5523-00
RNAscope 2.0 HD Reagent Kit-Brown	ACD	Cat#:320497
Mouse TCS Purification System	abcam	Cat#:ab128749
EZ-Link Sulfo-NHS-LC Biotinylation Kit	Thermo Fisher	Cat#:21435
Alexa Fluor 647 protein labeling kit	Thermo Fisher	Cat#:A20173
KAPA Stranded RNA-seq Kit with RiboErase (HMR)	Roche	Cat#:07962282001
LS-Columns	Mitlenyi Biotech	Cat#:130-042-401

**Sequence-Based Reagents**		

Primer sequences	See Table S1	N/A
Mu:Btnl1	Advanced Cell Diagnostics	Cat#:436641
Mu:Btnl4	Advanced Cell Diagnostics	Cat#:439811
Mu:Btnl6	Advanced Cell Diagnostics	Cat#:439821

**Deposited Data**		

RNA sequencing data	GEO: GSE85422	N/A

**Experimental Models: Cell Lines**		

MODE-K cells	Dr. D. Kaiserlian, INSERM-U1111; Bas et al., 2011	N/A

**Experimental Models: Organisms/Strains**		

B6.Cg-Foxn1 < Nu > /J (*nu/nu* mice)	The Jackson Laboratory, Stock: 000819	
Nur77.gfp mice	University of Minnesota, USA, K. Hogquist	N/A
*Btnl1*^*−/−*^ mice (Btnl1^tm1(KOMP)Mbp^)	IMPC, Project ID: CSD67994	N/A
*Btnl4*^*−/−*^ mice (Btnl4^tm1(KOMP)Mbp^)	IMPC, Project ID: CSD81524	N/A
Villin-rtTA2-M2 mice	Erasmus University, Rotterdam, M. Smits	N/A
*Btnl1*-Tg mice	This paper	N/A
*Btnl1*^*indel/indel*^	This paper	N/A
R26-rtTA2-M2 mice	Hochedlinger et al., 2005	N/A

**Recombinant DNA**		

Doxycycline-inducible CMV promoter plasmid pTRE2	Clontech	Cat#: 6241-1
Packaging plasmid pCMVΔR8.91	Zufferey et al., 1997	N/A
Packaging plasmid pHIT/G	Fouchier et al., 1997	N/A
Lentiviral vectors pCSIGPW, pCSIGHW	This paper	N/A
cDNA BTNL3 (GenBank: NM_197975.2), BTNL8S (Short, GenBank: NM_024850), BTNL8 (Long, GenBank: NM_001040462)	This paper	N/A
gRNA basic plasmid	Moises Mallo,Instituto Gulbenkian de Ciencia, Portugal	N/A
cDNA Btnl1, Btnl4, Btnl6	Bas et al., 2011	N/A

**Software and Algorithms**		

Imaris Software	Bitplane Scientific Solutions	http://www.bitplane.com/imaris/imaris
FlowJo (version 9)	FlowJo	http://www.flowjo.com/
Bowtie2	Johns Hopkins University	http://bowtie-bio.sourceforge.net/bowtie2/index.shtml

**Other**		

Cellfoam matrices	Cytomatrix PTY Ltd	Cat#: CY-903
Amino acid-containing (food-Ag-free diet)	ssniff	Cat#: S7242-E014/-E714

### Contact for Reagent and Resource Sharing

For additional information about reagents and resources, contact the Lead Contact, Adrian Hayday at adrian.hayday@kcl.ac.uk.

### Experimental Model and Subject Details

#### Mice

Wild-type (WT) C57Bl/6 mice were obtained from Charles River and Harlan. 3 independently derived embryonic stem (e.s.) cells for *Btnl1*^*−/−*^ (Btnl1^tm1(KOMP)Mbp^) and e.s. cells for *Btnl4*^−/−^ (Btnl4^tm1(KOMP)Mbp^) mice were obtained from the international mouse phenotyping consortium (IMPC) (project IDs: CSD67994 and CSD81524). *Btnl1*^indel/indel^ mice were generated using CrisprCas Technology. Briefly: Two independent short guide RNAs, targeting the intronic region: between exon1 and 2 and between exon 5 and 6 were identified using the online tool: http://crispr.mit.edu/.

Intron1/2: CCAGCTCCAAGATCCCCCTTGGG Intron5/6: TCCATAGCACCTTATCCGGTTGG

The sg RNAs & PAM sequences were cloned into the g-RNA basic vector, translated in vitro, purified and co-injected with Cas9 into day 1 zygotes and transferred into pseudopregnant foster mice.

WT and *Btnl*-knockout lines were generated and maintained at The Francis Crick Institute’s Biological resource facilities. B6.Cg-Foxn1 < Nu > /J (NU/NU) mice were obtained from the Jackson Laboratory. Nur77.gfp mice were kindly provided by K. Hogquist (Moran et al., 2011). For timed pregnancies, mice were mated overnight and E0 was considered as the day a vaginal plug is observed. Both male and female mice aged between 1 and 35 weeks (as indicated) were used in this study. No gender-specific differences were observed.

#### Germ-free Mice and Food Antigen-free Nutrition

C57Bl/6 mice maintained on germ-free or on solid-food antigen-free diets were bred at the Institute for Medical Microbiology and Hospital Epidemiology, University of Marburg, Germany where all experiments were conducted according to the German animal protection law.

Germ-free (GF) mice were kept in plastic isolators (Metall and Plastik, Germany) with autoclaved food, bedding and water. Sterility of animals was checked bi-weekly by culturing faeces in thioglycollate medium under aerobic and anaerobic conditions for at least ten days. All handling procedures for GF mice were conducted in a laminar flow hood under sterile conditions. All experiments were conducted according to the German animal protection law.

Food antigen-free (FAF) mice were raised on an amino acid-containing diet for up to five generations. Pellets of FAF diet (ssniff, S7242-E014/-E714) contained all essential vitamins, minerals, trace elements, fat, dextrin, sucrose and free amino acids equimolar to the protein content of normal rodent chow (LASQCdietRod16, LASvendi).

#### Generation of Doxycycline Inducible Btnl-1 Transgenic Mice

Doxycycline (Dox)-inducible *Btnl1*-Tg mice were generated by injection of *Btnl1*^−/−^ blastocysts with a linearized cassette containing a TRE/CMV-promoter upstream of the Btnl1-ORF. The TRE/CMV cassette has been previously described (Oppenheim et al., 2005). R26-rtTA2-M2 (Hochedlinger et al., 2005) or Villin-rtTA2-M2 (Roth et al., 2009) mice were bred to homozygosity for *Btnl1*-deficiency and backcrossed onto *Btnl-*Tg mice for 3 generations to facilitate global (R26) or local (Villin) induction of *Btnl1* transgene expression by doxycycline administered to drinking water (1mg/ml Dox, 2% suchrose). Animal experiments were undertaken in full compliance with UK Home Office regulations and under a project license to A.H. (80/2480).

#### Flow Cytometry

Flow cytometry was performed using the following antibodies, coupled to the indicated fluorochromes (see key resources table). *Antibodies for mouse:* CD3 APC Cy7 (17A2); CD3 PerCPCy5.5 (145-2C11); TCRβ Brilliant Violet 421 (H57-597); TCRβ APC (H57-597); CD122 PE (TMβ1); CD122 Brilliant Violet 421 (TMβ1); CD122 APC (TMβ1); TIGIT PE (GIGD7); CD45RB APC Cy7 (C363-16A); Thy1.2 Brilliant Violet 510 (53-2.1); Lag3 PerCP-efluor 710 (C9B7W); CD5 PE (53-7.3); CD24 FITC (M1/69); CD24 PECy7 (M1/69); CD8α PECy7 (53-6.7); CD8α PECy7 (53-6.7); TCR Vδ4 FITC (GL-2); TCR Vδ4 PE (GL-2); CD8β PerCpCy5.5 (YTS156.7.7); CD25 PerCpCy5.5 (PC61); CD69 PECy7 (H1.2F3); CCR9 PECy7 (CW-1.2); CD44 PECy7 (IM7); TCRVγ7 (F2.67) was provided by Pablo Pereira (Institut Pasteur, Paris, France); TCRVγ1 APC (2.11); TCRVγ4 APC (UC3-10A6); TCRδ BV421 (GL3); Ki67 FITC (B56/MOPC-21); CD45 Qdot 605 (30-F11); CD5 Brilliant Violet 510 (53-7.3); TCRδ PeCy7 (GL3); CD161/NK1.1 Brilliant Violet 650 (PK136); CD4 Brilliant Violet 786 (GK1.5); CD8α AlexaFluor 700 (53-6.7); CD25 APC (PC61); GITR PE (DTA-1); CD44 FITC (IM7); CD62L PerCP-Cy5.5 (MEL-14); KLRG1 BV421 (2F1); CD11c BV786 (HL3); CD11b BV510 (M1/70); F4/80 PerCPCy5.5 (BM8); Ly6G APC (1A8); Ly6C AlexaFluor 700 (AL-21); CD103 PE (M290); CD317 Brilliant Violet 650 (927); MHCII/IA/IE FITC (2G9); CD86 Pe-Cy7 (GL1); CD3 Brilliant Violet 421 (145-2C11); CD19 Brilliant Violet 421 (1D3); CD161/NK1.1 (lin) Brilliant Violet 421 (PK136); IgG1 PE (A85-1); B220 (CD45R) AlexaFluor 700 (RA3-6B2); IgM Brilliant Violet 786 (R6-60.2); IgD PerCPCy5.5 (11-26c.2a); GL-7 AlexaFluor 647 (GL7); CD95 PECy7 (Jo2); CD138 Brilliant Violet 650 (281-2); CD21/35 FITC (7G6); CD23 Brilliant Violet 421 (B-ly6). *Antibodies for human:* CD25 Brilliant Violet 421 (BC96); CD25 PE (BC96); CD3 Brilliant Violet 510 (OKT3); CD3 BUV (UCHT 1); EpCAM eFlour 660 (1B7); Streptavidin APC-Cy7; Streptavidin Brilliant Violet 421; TCRγδ PeCy7 (IMMU510); Vγ9 PC5 (IMMU360); Vγ9 PE (B3); Vδ1 APC (REA173); Vδ2 PerCP (B6); Vγ2/3/4 biotin (23D12), Vγ3/5 biotin (56.3) and Vγ8 biotin (R4.5.1) were provided by D. Kabelitz and D. Wesch (University of Kiel). *Other antibodies:* DYKDDDDK-PE (Flag); DYKDDDDK-APC (Flag); HA-DyLight 650; 6x-Histidine-PE.

Commercial antibodies were purchased from Biolegend, eBioscience, BD-Bioscience, Thermo Fisher Scientific or Miltenyi (see key resource Information). Viability dyes (near IR or Blue) were from Invitrogen. Anti TCRVγ7 (F2.67) was purified from hybridoma supernatant using the mouse TCS purification system (Abcam) and conjugated to biotin or AF647 (see key resources table).

Ki-67 staining was performed on cells fixed and permeabilised using the Foxp3 staining buffer set (eBioscience). BrdU (Sigma-Aldrich) and EdU incorporation was assessed 3h post-intraperitoneal injection (50mg/kg) by immunohistochemistry or by flow cytometry (Click-iT EdU AF647 Assay Kit, Invitrogen), respectively.

Anti-TCRVγ7 (F2.67, provided by Pablo Pereira [Insitut Pasteur, Paris, France]) was purified from hybridoma supernatant using the mouse TCS purification system (abcam-ab128749). Purified anti-TCRVγ7 was conjugated to biotin (EZ-Link Sulfo-NHS-LC Biotinylation Kit, Thermo Fisher Scientific) or to AF647 (labeling kit, Thermo Fisher Scientific). Anti-human Vγ2/3/4 (23D12, biotinylated), Vγ5/3 (56.3, biotinylated) and Vγ8 (R4.5.1, biotinylated) were provided by D. Kabelitz & D.Wesch (Institute of Immunology, Kiel, Germany).

Flow cytometry data analysis was performed on FlowJo (Version 9.9).

#### Plasmids, Cloning, RT-PCR, Transfection and Lentiviral Transduction

The self-inactivating lentiviral vector pCSIGPW (SFFV promoter – Multiple Cloning Site [MCS] – IRES-GFP – CMV promoter – Puromycin^R^) was constructed by replacing the Puromycin^R^/mIR cassette from the pAPM vector (Pertel et al., 2011) by a custom EcoRI-XhoI-PmeI-NotI-BamHI-XbaI-MluI MCS. The IRES-GFP cassette was cloned by PCR from the pIRES2-eGFP vector (Clonetech) using the BamHI/XbaI sites. The CMV promoter was cloned by PCR from the pCDNA3.1+ vector (Thermo Fischer Scientific) using the MluI/ClaI sites. The Puromycin resistance gene was cloned by PCR from the pGIPZ vector (Dharmacon) using the ClaI/AgeI sites. The pCSIGHW variant was generated by exchanging the puromycin resistance gene with a hygromycin B resistance gene, which was cloned by PCR from the pLHCX vector (Clontech).

cDNAs were (sub-)cloned into pCSIGPW or variant vectors (see Supplemental Information). *Btnl1*, *Btnl4* and *Btnl6* were previously described (Bas et al., 2011). *BTNL3* (GenBank:NM_197975.2), *BTNL8S* (GenBank:NM_024850), and *BTNL8* (GenBank:NM_001040462) were cloned from Caco-2 cells by conventional RT-PCR, using the following primers (See Table S2):

BTNL3 For 5′-GAATATCCATGGCTTTTGTGC-3′
BTNL3 Rev 5′-GTCTTCTCTGTCTCATCCCC-3′
BTNL8 For 5′-CCATTCACAGAACACATCCATG-3′
BTNL8S Rev 5′-TATGGGTTACAGTTTTCAGATCAG-3′
BTNL8 Rev 5′-GTGGGATGTGATTCATCCTAC-3′

FLAG, HA and HIS tags were added downstream of the putative leader peptides by overlapping PCR. Human full-length TCR γ and δ chains were cloned (XhoI / NotI, pCSIGPW) using the following primers (See Table S2):

Vγ2/3/4 For 5′-ATGCAGTGGGCCCTAGCG-3′
Vγ8 For 5′-ATGCTGTTGGCTCTAGCTCTGCTTC-3′
Vγ9 For 5′-ATGCTGTCACTGCTCCACACATC-3′
Cγ1/2 Rev 5′-TTATGATTTCTCTCCATTGCAGCA G-3′
Vδ1 For 5′-ATGCTGTTCTCCAGCCTGCTG-3′
Vδ2 For 5′-ATGCAGAGGATCTCCTCCCTCAT-3′
Vδ3 For 5′-ATGATTCTTACTGTGGGCTTTAGCTTTTTG-3′
Cδ Rev 5′-TTACAAGAAAAATAACTTGGCAGTCAAGAG-3′

Expression of *BTNL3* and *BTNL8* was checked by conventional RT-PCR using the primers indicated above. *BTN3A1*, *BTN3A2*, *EPCAM* and *GAPDH* were used as control genes (See Table S2):

BTN3A1 For 5′-AGTATCTCCTGATATGCAGCATG-3′
BTN3A1 Rev 5′-GGAGGAACTCTCTTCTTCTTTTCAC-3′
BTN3A2 For 5′-TGGTATCTCTTGATATGCAGCATAG-3′
BTN3A2 Rev 5′-AGAGCATCAGGCTGACTTATTGG-3′
EPCAM For 5′-GCCGCCACCATGGCGCCCCCGCAG-3′
EPCAM Rev 5′-TTATGCATTGAGTTCCCTATGCA-3′
GAPDH For 5′-GAAGGTGAAGGTCGGAGTC-3′
GAPDH Rev 5′-GAAGATGGTGATGGGATTTC-3′

Transfections were carried out in HEK293T cells using PEI (3:1 PEI:DNA ratio, Polysciences). Btnl/BTNL expression was checked 48h post-transfection. Lentiviral particles were produced in HEK293T cells by co-transfection of pCSIGPW or pCSIGHW either empty or containing *Btnl/BTNL* cDNAs, pCMVΔR8.91 (HIV-1 *tat*/*rev*/*gag*/*pol*) (Zufferey et al., 1997), and pHIT/G (MLV *env*) (Fouchier et al., 1997). Transduced cells were treated with puromycin and hygromycin 48h post-transduction for 7 days, sorted on the basis of GFP expression and used for functional assays.

#### Quantitative RT–PCR

Samples were stored in RNAlater (Ambion) or directly frozen in RLT buffer prior to RNA purification (QIAGEN RNeasy kit). cDNA was generated using Superscript-II (Invitrogen) and analyzed using Sybr-green assay (Invitrogen) using a ViiA7 Real-time PCR machine (Applied Biosystems) (See Table S2).

#### Primers for Murine qPCR

Btnl1 For: 5′-TGACCAGGAGAAATCGAAGG-3′
Btnl1 Rev: 5′-CACCGAGCAGGACCAATAGT-3′
Btnl4 For: 5′-CATTCTCCTCAGAGACCCACACTA-3′
Btnl4 Rev: 5′-GAGAGGCCTGAGGGAAGAA-3′
Bntl6 For: 5′-GCACCTCTCTGGTGAAGGAG-3′
Btnl6 Rev: 5′-ACCGTCTTCTGGACCTTTGA-3′
β-Actin For: 5′-CAGCTTCTTTGCAGCTCCTT-3′
β-Actin Rev: 5′-CACGATGGAGGGGAATACAG-3′
Sox-13 For: 5′-CTCCAGGCCTTCCCAGAC-3′
Sox-13 Rev: 5′-CATGGACTTCCAGCGAGAAC-3′
Rorγc For: 5′-GGTGACCAGCTACCAGAGGA-3′
Rorγc Rev: 5′-CCACATACTGAATGGCCTCA-3′
Tbp For: 5′-GGGGAGCTGTGATGTGAAGT-3′
Tbp Rev: 5′-CCAGGAAATAATTCTGGCTCA-3′
CycloFor: 5′-CAAATGCTGGACCAAACACAA-3′
Cyclo Rev: 5′-CCATCCAGCCATTCAGTCTTG-3′

#### Southern Blotting

Southern blots were performed with probes generated using a Dig-Probe labeling kit; blots were hybridized in DIG-Easy-hyb buffer overnight, and developed using the DIG-Luminescence Detection Kit (Sigma-Aldrich). For probe sequences see Supplementary Information. DIG labeled probes for Southern blotting were generated using the following primers (See Table S2):

Btnl1 For: 5′-ACTGGCTTCCTCAGAGTCAT-3′
Btnl1 Rev: 5′-CAGTAGTGAATGGCCCCTGA-3′
Btnl4 For: 5′-GACCAACGCTTCCCTACCTC-3′
Btnl4 Rev: 5′-GCCTTGGGTCCAACAAGACA-3′
Btnl1-Tg-Ex3-For: 5′-GGTTTTCTGTGAAGGGACCA-3′
Btnl1-Tg-Ex4-Rev: 5′-GGTCTGCAACTCAGAGGAGG-3′

#### RNAscope

RNAscope was performed on paraffin embedded sections using probes and kits obtained from Advanced Cell Diagnostics using the RNAscope 2.0 HD Reagent Kit-BROWN. Reference sequences are as follows: *Btnl1*, GenBank:NM_001111094.1 (576-1723); *Btnl4*, GenBank:NM030746.1 (560-968); *Btnl6*, GenBank:NM_030747.1 (245-1552) (See Table S2).

#### Isolation of Murine Intestinal Intra-epithelial Lymphocytes (IEL)

IEL were isolated from mouse small intestine as previously described (Wencker et al., 2014). Small intestine was opened and washed in PBS, cut into 1cm pieces and incubated for 20min in RPMI 1640 supplemented with 1% penicillin/streptomycin (pen/strep), 10% fetal calf serum (FCS) and 1mM dithiothreitol on a turning wheel. Tissues were washed and vortexed in RPMI, then passed through a 70 μm nylon cell strainer twice, and centrifuged on a 20/40/80% Percoll density gradient at 700 g for 30min. IEL were harvested from the 40 to 80% Percoll interface.

#### Spleen and Mesenteric Lymph Node Immunophenotyping

Comprehensive immunophenotyping of Btnl1-/- mice was performed using a platform developed by the Wellcome Trust Infection and Immunity Immunophenotyping (3i) consortium (www.immunophenotyping.org). In brief, Spleen and MLN were digested with collagenase (1mg/ml)/DNAse (0.1 mg/ml) in 2% FCS PBS (+ Ca/Mg) for 20 minutes at 37°C and filtered through 30μm cell strainers. Cells were plated on 96 well V-bottom plates, washed in PBS and stained with Zombie Near-IR (Biolegend) for live/dead discrimination. Antibody stains were performed at 4°C for 20mins. Full details regarding phenotyping panels are included in Table S3. Samples were acquired on a BD LSR Fortessa X-20 equipped with 405nm (40mW), 488nm (50mW), 561nm (50mW), and 640nm (100mW) lasers.

#### MODE-K Co-culture Assays

Cells were co-cultured in RPMI 1640 supplemented with 10% FCS, Pen/Strep, 2.5% HEPES, 1% Glutamine, 1% non-essential amino acids, 1% sodium pyruvate, 0.2% β-mercapto-ethanol (GIBCO) and cytokines including IL-2 (10U/ml), IL-15 (10 ng/ml) (Immunotools), IL-3 (100U/ml), IL-4 (200U/ml) (R&D). 10^5^ MODE-K were seeded in 48-well plates 24h prior to the addition of 10^5^ unsorted or (where indicated) positively FACS-sorted (CD45+Vγ7+) IEL and incubated for 16-18h in 10% CO_2_ unless indicated otherwise. For transwell assays, 2x10^5^ MODE-K cells were seeded onto 24-well transwell plates (3 μm pore size - Corning) 24h prior to the addition of 3x10^5^ IEL, either in direct contact (below), sequestered from (above), or split 50:50 with MODE-K cells (above and below the transwell).

#### IEL Stimulation

96-well U bottom plates were coated overnight with 10 μg/ml LEAF-Purified anti-mouse CD3-ε or Hamster IgG Isotype control (Biolegend) at 4°C and washed once with PBS 1x before seeding IEL. 100,000 IEL were seeded per well. Cells were incubated at 37°C for 16-18h in 10% CO_2_ prior to analysis.

#### Confocal Imaging

Proximal small intestine (SI) samples were fixed in Zamboni’s fixative, blocked with normal goat serum and stained with antibodies against TCRβ, TCRδ, TCRVδ4 (encoded by TRDV2-2) (GL2), CD3 and Vγ7. Z-Sections were acquired on a confocal-LSM-710 microscope (Zeiss) and processed and analyzed using Imaris Software (Bitplane Scientific Solutions).

#### Bone Marrow Chimeras and Adoptive IEL Transfers

10-12 week old recipient mice were irradiated with 950Rads 24h, injected (IV) with 5-10x10^6^ donor bone marrow cells and analyzed 4-12 weeks later.

IEL harvested from 4 week-old WT mice were column-purified using CD45 microbeads (MACS Miltenyi biotec) and IV-injected into 6 week-old TCRδ^−/−^ and TCR**δ**^−/−^*Btnl1*^−/−^ recipients. Analysis was performed 2-3 weeks later.

#### RNA Sequencing

Vγ7^+^CD122^hi^ and Vγ7^+^CD122^lo^ IEL were sorted from from pooled D14-17 pups directly into RLT buffer. RNA was prepared using the RNA-Micro-plus kit (QIAGEN). RNA libraries were generated using the KAPA Stranded RNA-seq Kit with RiboErase (HMR) (KAPA BIOSYSTEMS). Paired-end sequencing on HiSeq 2500 (illumina) using rapid run chemistry (read length: 100bp).

#### Human Samples and Primary Lymphocyte Isolation

Endoscopic biopsies were obtained from the ascending colon of adult donors undergoing routine diagnostic colonoscopy after informed consent and in compliance with local ethical approval (REC number 07/H0803/237). Excess resected skin discarded at the time of cutaneous or reconstructive surgery was obtained from adult donors after informed consent and in compliance with local ethical approval (REC number 06/Q0704/18). This study was conducted adhering to the principles of the Declaration of Helsinki.

Primary gut lymphocytes were obtained using an adaptation of the method of Kupper and Clarke (Clark et al., 2006) (Figure S7F). Skin lymphocytes were isolated using the method as originally described (Clark et al., 2006). 9mm x 9mm x 1.5mm Cellfoam matrices (Cytomatrix PTY Ltd), were autoclaved and incubated in 100mg/mL rat tail collagen I (BD Biosciences) in PBS for 30min at 37°C, and washed twice in PBS. In compliance with local ethical approval, 12 endoscopic biopsies were taken from the ascending colon of donors. Biopsies were washed for 20min in 5mL wash medium (RPMI 1640 10% FCS, β-mercaptoethanol, penicillin [500U/ml], streptomycin [500 μg/ml], metronidazole [5 μg/ml, Pharmacy department, Guy’s Hospital], gentamicin [100 μg/ml, Sigma-Aldrich] and amphotericin 12.5 μg/ml [Thermo Fisher Scientific]). One endoscopic biopsy was placed on top of each matrix, which was inverted, and pressure applied, to crush the biopsy into the matrix. The matrices were placed into a 24-well plate (1 per well) and covered with 2mL RPMI 1640 (supplemented with 10% FCS, β-mercaptoethanol, penicillin [100U/ml], streptomycin [100 μg/ml], metronidazole [1 μg/ml], gentamicin [20 μg/ml], amphotericin [2.5 μg/ml]), IL-2 (100U/mL, Novartis Pharmaceutical UK) and IL-15 (10ng/mL, Biolegend). 1 ml of medium was aspirated every second day and replaced with complete medium containing 2x concentrated cytokines. Cells were harvested and residual biopsy and empty wells were washed with PBS 0.02mM HEPES. The cell suspension was passed through a 70 μm nylon cell strainer, centrifuged at 400 g for 5min and resuspended in complete medium without additional cytokine and placed into co-culture immediately. Lymphocytes were used after 5-7 days of culture.

PBMC were isolated by Ficoll gradient from blood obtained from the blood donation service.

#### Human Epithelial Cell Isolation

Colonic samples were incubated with 5 mM 1,4-dithiothreitol (Sigma), followed by enzymatic digestion with 1.5mg/ml collagenase VIII (Sigma) and 0.05 mg/mL DNase I (Sigma). EpCAM^+^ cells were sorted by flow cytometry directly into RLT lysis buffer. RNA and cDNA were prepared as described above.

#### HEK293T Co-culture Assay

5x10^5^ HEK293T cells, transduced with either empty vector (EV), BTNL3, BTNL8 or BTNL3+8 and 2x10^5^ freshly harvested primary human lymphocytes were co-cultured in 96-well plates with complete medium (see Supplementary Information) without supplementary cytokine and incubated at 37°C at 5% CO2 for 16hrs (Figure S7F).

#### Deep Sequencing

Mouse TRDV gene: Amplification and sequencing of TCRδ CDR3 from RNA purified from sorted Vγ7^+^ IEL was performed using the Amp2Seq Platform (iRepertoire).Human TCRG Vγ gene: Amplification and sequencing of TCRγ CDR3 was performed using the immunoSEQ Platform (Adaptive Biotechnologies).

### Quantification and Statistical Analysis

#### Statistics

Unless stated otherwise, bar/spider charts display mean ± SD and p values were derived from unpaired two tailed t tests, assuming equivalent SD (*ns* > 0.05).

#### Imaris Image Analysis

Confocal microscopy was performed using a LSM710 laser scanning confocal microscope (Zeiss) with a 40x oil objective (numerical aperture 1.3). 3D image analysis on z-stacks was carried out using Imaris (Bitplane). The surfaces tool was used to identify CD3^+^ cells. Voxels outside of these structures were set to zero in each of the channels to create masks.

#### Bioinformatics Analysis of RNA Sequencing

101 base-pair paired-end reads were aligned and quantified using RSEM (v1.2.11) (Li and Dewey, 2011) with Bowtie2. Reads were aligned to a transcriptome constructed from the mm10 mouse genome and a UCSC knownGene gtf file. A mean alignment rate of 57.4 million fragments per sample was observed. Using the gene level quantification, only detected genes (mean TPM value across all samples > 1; 13,313 genes) were selected. Differential expression between the CD122^hi^ and CD122^lo^ Vγ7^+^ IEL groups using DESeq2 (Love et al., 2014) was identified by taking into account the paired structure within the replicate groups. Using an FDR of 0.01 2664 phenotype dependent gene expression effects were identified.

### Data and Software Availability

#### Data Resources

The accession number for the RNA sequencing data reported in this paper is GEO: GSE85422.

## Author Contributions

Conceptualization, A.H.; Methodology, A.H., A.J., L.D., R.D.M.B., P.V., R.J.D., O.N., U.S., and R.H.; Resources, D.G., P.M.I., P.P., and U.S.; Investigation, R.D.M.B., N.A.R., R.J.D., P.V., A.J., O.N., L.D., S.C., R.H., M.L.I., A.L., B.S.-D., P.E., and U.S.; Validation, R.J.D., L.D., and A.J.; Data Curation, R.D.M.B., B.S.-D., P.E., P.V., and A.H.; Writing – Original Draft, A.H.; Writing – Review & Editing, A.H., U.S., R.D.M.B., N.A.R., R.J.D., P.V., A.J., L.D., P.P., and S.C.; Visualization, R.D.M.B., N.A.R., R.J.D., A.J., R.H., B.S-D., P.V., and A.L.; Supervision, A.H.; Funding Acquisition, U.S. and A.H.
