# Supplementary material for: Epithelia Use Butyrophilin-like Molecules to Shape Organ-Specific γδ T Cell Compartments
Source: Cell. 2016 Sep 22;167(1):203–218.e17. doi: 10.1016/j.cell.2016.08.030 (PMC5037318; doi:10.1016/j.cell.2016.08.030)
Supplement: Document S1. Tables S1–S3 [file mmc1.pdf]

Cell, Volume *167*

Supplemental Information

Epithelia Use Butyrophilin-like Molecules

to Shape Organ-Specific $\gamma\delta$ T Cell Compartments

Rafael Di Marco Barros, Natalie A. Roberts, Robin J. Dart, Pierre Vantourout, Anett Jandke, Oliver Nussbaumer, Livija Deban, Sara Cipolat, Rosie Hart, Maria Luisa Iannitto, Adam Laing, Bradley Spencer-Dene, Philip East, Deena Gibbons, Peter M. Irving, Pablo Pereira, Ulrich Steinhoff, and Adrian Hayday

Table S1

A

	Vδ1	Vδ2	Vδ1⁻Vδ2⁻
Vγ2/3/4 (n=10) % of	60.9 (19.5)	2.5 (3.1)	64.7 (18.7)
Vγ3/5 (n=6) % of	13.1 (10.9)	1.4 (1.4)	24.5 (25.8)
Vγ8 (n=7) % of	37.7 (28.0)	0.9 (1.0)	14.4 (12.0)
Vγ9 (n=7) % of	9.3 (16.0)	88.5 (17.1)	9.3 (11.2)

B

```
                                                       V                         N,P      J       C
G234GU70.1 (V4-J1-C1)    SGISPGKYDTYGSTRKNLRMILRNLIENDSGVYYCATWDGPGYKKLFGSGTTLVV--TDK
G234GU70.2 (V4-J1-C1)    SGISPGKYDTYGSTRKNLRMILRNLIENDSGVYYCATWDGPGYKKLFGSGTTLVV--TDK
G234GU70.3 (V4-J1-C1)    SGISPGKYDTYGSTRKNLRMILRNLIENDSGVYYCATWDGQGDKKLFGSGTTLVV--TDK
G234GU70.4 (V4-J2-C2)    SGISPGKYDTYGSTRKNLRMILRNLIENDSGVYYCATWDAPN-KKLFGSGTTLVV--TDK
G234GU70.5 (V4-JP2-C2)   SGISPGKYDTYGSTRKNLRMILRNLIENDSGVYYCATWEAG-WFKIFAEGTKLIVTSPDK
G234SK01.1 (V2-J2-C2)    SGVSPGKYYTYASTRNNLRLILRNLIENDSGVYYCATWIS--YKKLFGSGTTLVV--TDK
G234SK01.2 (V3-J1-C1)    SGLSPGKYYTHTPRRWSWILRLQNLIENDSGVYYCATWDP-YYKKLFGSGTTLVV--TDK
G234SK01.3 (V3-J1-C1)    SGLSPGKYYTHTPRRWSWILRLQNLIENDSGVYYCATWDP-YYKKLFGSGTTLVV--TDK
G234SK01.4 (V3-J1-C1)    SGLSPGKYYTHTPRRWSWILRLQNLIENDSGVYYCATWDGLR--KLFGSGTTLVV--TDK
G234SK01.5 (V3-J1-C1)    SGLSPGKYYTHTPRRWSWILRLQNLIENDSGVYYCATWDGLR--KLFGSGTTLVV--TDK
                         **:***** *:   *  .  : *:****************      *:*..**.*:*   **
```

Table S2

Target (RT-PCR)	Forward	Reverse
Hu: BTNL3 primers	GAATATCCATGGCTTTTGTGC	GTCTTCTCTGTCTCATCCCC
Hu: BTNL8 Short	CCATTCACAGAACACATCCATG	TATGGGTTACAGTTTTCAGATCAG
Hu: BTNL8 Long	CCATTCACAGAACACATCCATG	GTGGGATGTGATTCATCCTAC
Hu: TCR Vγ2/3/4	ATGCAGTGGGCCCTAGCG	N/A
Hu: TCR Vγ8 (Fw)	ATGCTGTTGGCTCTAGCTCTGCTT C	N/A
Hu: TCR Vγ9 (Fw)	ATGCTGTCACTGCTCCACACATC	N/A
Hu: TCR Cγ1/2 (Rv)	N/A	TTATGATTTCTCTCCATTGCAGCA
Hu: TCR Vδ1 (Fw)	ATGCTGTTCTCCAGCCTGCTG	N/A
Hu: TCR Vδ2 (Fw)	ATGCAGAGGATCTCCTCCCTCAT	N/A
Hu: TCR Vδ3 primer (Fw)	ATGATTCTTACTGTGGGCTTTAGC TTTTTG	N/A
Hu: TCR Cδ (Rv)	N/A	TTACAAGAAAAATAACTTGGCAGT CAAGAG
Hu: BTN3A1	AGTATCTCCTGATATGCAGCATG	GGAGGAACTCTCTTCTTCTTTTCA C
Hu: BTN3A2	TGGTATCTCTTGATATGCAGCATA G	AGAGCATCAGGCTGACTTATTGG
Hu: EPCAM	GCCGCCACCATGGCGCCCCCGC AG	TTATGCATTGAGTTCCCTATGCA
Hu: GAPDH	GAAGGTGAAGGTCGGAGTC	GAAGATGGTGATGGGATTTC
**Target (qRT-PCR)**	**Forward**	**Reverse**
Mu: Btn1	TGACCAGGAGAAATCGAAGG	CACCGAGCAGGACCAATAGT
Mu: Btnl4	CATTCTCCTCAGAGACCCACACTA	GAGAGGCCTGAGGGAAGAA
Mu: Btnl6	GCACCTCTCTGGTGAAGGAG	ACCGTCTTCTGGACCTTTGA
Mu: β-Actin	CAGCTTCTTTGCAGCTCCTT	CACGATGGAGGGGAATACAG
Mu: Sox13	CTCCAGGCCTTCCCAGAC	CATGGACTTCCAGCGAGAAC
Mu: Rorc	GGTGACCAGCTACCAGAGGA	CCACATACTGAATGGCCTCA
Mu: Tbp	GGGGAGCTGTGATGTGAAGT	CCAGGAAATAATTCTGGCTCA
Mu: Cyclophilin	CAAATGCTGGACCAAACACAA	CCATCCAGCCATTCAGTCTTG
**Target (primers to generate Southern probes)**	**Forward**	**Reverse**
Mu: Btnl1	ACTGGCTTCCTCAGAGTCAT	CAGTAGTGAATGGCCCCTGA
Mu:Btnl4	GACCAACGCTTCCCTACCTC	GCCTTGGGTCCAACAAGACA
Mu: Btnl1-Tg	GGTTTTCTGTGAAGGGACCA	GGTCTGCAACTCAGAGGAGG

Table S3

Population	Description by markers	Lineage
Total T cells	CD45+ , CD5+ , TCRδ+ or CD4+ or CD8+	T
Total γδ T cells	CD45+ , TCRδ+,	T
Effector γδ T cells	CD45+ , TCRδ+ , CD44+ , CD62L-	T
Resting γδ T cells	CD45+ , TCRδ+ , CD62L+	T
KLRG1+ γδ T cells	CD45+ , TCRδ+ , KLRG1+	T
CD5+ γδ T cells	CD45+ , TCRδ+ , CD5+	T
Total αβ T cells	CD45+ , CD5+ , TCRδ- , CD4+ or CD8+	T
Total CD8+ T cells	CD45+ , CD5+ , TCRδ- , CD4- , CD8+	T
Effector CD8+ T cells	CD45+ , CD5+ , TCRδ- , CD4- , CD8+ , CD44+, CD62L-	T
Resting CD8+ T cells	CD45+ , CD5+ , TCRδ- , CD4- , CD8+ , CD44+ , CD62L+	T
Naive CD8+ T cells	CD45+ , CD5+ , TCRδ- , CD4- , CD8+ , CD44- , CD62L+	T
KLRG1+ CD8+ T cells	CD45+ , CD5+ , TCRδ- , CD4- , CD8+, KLRG1+	T
Total CD4+ T cells	CD45+ , CD5+ , TCRδ- , CD4+ , CD8-	T
CD4+ T helper cells	CD45+ , CD5+ , TCRδ- , CD4+ , CD8- , CD25- , GITR-	T
Effector CD4+ T helper cells	CD45+ , CD5+ , TCRδ- , CD4+ , CD8- , CD25- , GITR- , CD44+ , CD62L-	T
Resting CD4+ T helper cells	CD45+ , CD5+ , TCRδ- , CD4+ , CD8-, CD25- , GITR- , CD62L+	T
KLRG1+ CD4+ T helper cells	CD45+ , CD5+ , TCRδ- , CD4+ , CD8- , CD25- , GITR- , KLRG1+	T
Total Tregs	CD45+ , CD5+ , TCRδ- , CD4+ , CD8- , CD25+ , GITR+	T
Effector Tregs	CD45+ , CD5+ , TCRδ- , CD4+ , CD8- , CD25+ , GITR+ , CD44+ , CD62L-	T
Resting Tregs	CD45+ , CD5+ , TCRδ- , CD4+ , CD8- , CD25+ , GITR+ , CD62L+	T
KLRG1+ Tregs	CD45+ , CD5+ , TCRδ- , CD4+ , CD8- , CD25+ , GITR+ , KLRG1+	T
Total NKT cells	CD45+ , CD5+ , TCRδ- , CD161+	NKT
Total CD4- NKT cells	CD45+ , CD5+ , TCRδ- , CD161+ , CD4-	NKT
Effector CD4- NKT cells	CD45+ , CD5+ , TCRδ- , CD161+ , CD4- , CD44+ , CD62L-	NKT
Resting CD4- NKT cells	CD45+ , CD5+ , TCRδ- , CD161+ , CD4- , CD62L+	NKT
KLRG1+ CD4- NKT cells	CD45+ , CD5+ , TCRδ- , CD161+ , CD4- , KLRG1+	NKT
Total CD4+ NKT cells	CD45+ , CD5+ , TCRδ- , CD161+ , CD4+	NKT
Effector CD4+ NKT cells	CD45+ , CD5+ , TCRδ- , CD161+ , CD4+ , CD44+ , CD62L-	NKT
Resting CD4+ NKT cells	CD45+ , CD5+ , TCRδ- , CD161+ , CD4+ , CD62L+	NKT
KLRG1+ CD4+ NKT cells	CD45+ , CD5+ , TCRδ- , CD161+ , CD4+ , KLRG1+	NKT
Total NK cells	CD45+ , CD5- , TCRδ- , CD4- , CD8- , CD161+	NK
Effector NK cells	CD45+ , CD5- , TCRδ- , CD4- , CD8- , CD161+ , CD44+ , CD62L-	NK
Resting NK cells	CD45+ , CD5- , TCRδ- , CD4- , CD8- , CD161+ , CD62L+	NK
KLRG1+ NK cells	CD45+ , CD5- , TCRδ- , CD4- , CD8- , CD161+ , KLRG1+	NK
Total B cells	CD45+ ,B220+ , CD138-	B
B1a cells	CD45+ ,B220+, CD138- , CD5+	B
Total B2 cells	CD45+ ,B220+, CD138- , CD5-	B
Marginal zone B cells	CD45+ ,B220+, CD138- , CD5- , GL-7- , CD95- , IgM+ , IgG- , CD21high , CD23+	B
Marginal zone precursor B cells	CD45+ ,B220+, CD138- , CD5- , GL-7- , CD95- , IgM+ , IgG- , CD21high , CD23-	B
Transitional 1 B cells	CD45+ ,B220+, CD138- , CD5- , GL-7- , CD95- , IgM+ , IgG- , CD21- , CD23-	B
Transitional 2 B cells	CD45+ ,B220+, CD138- , CD5- , GL-7- , CD95- , IgM+ , IgG- , CD21- , CD23+	B
Follicular B cells	CD45+ ,B220+, CD138- , CD5- , GL-7- , CD95- , IgM+ , IgG- , CD21low , CD23+	B
Plasma cells	CD45+ B220low , CD138+	B
Memory B cells	CD45+ ,B220+, CD138- , CD5- , GL-7- , CD95- , IgM- , IgG+	B
Germinal centre B cells	CD45+ ,B220+, CD138- , CD5- , GL7+ , CD95+	B
Early germinal centre B cells	CD45+ ,B220+, CD138- , CD5- , GL7+ , CD95+ , IgM+ , IgG-	B
Late germinal centre B cells	CD45+ ,B220+, CD138- , CD5- , GL7+ , CD95+ , IgM- , IgG+	B
Total myeloid cells	CD19- , CD3-, NK1.1-,	Myeloid
Macrophages	CD19- , CD3-, NK1.1-, F4/80+ , CD11blow	Myeloid
Granulocytes	CD19- , CD3-, NK1.1-, F4/80- , Ly6C+ , Ly6G+ , CD11b+	Myeloid
Monocytes	CD19- , CD3-, NK1.1-, F4/80- , Ly6C+ , Ly6G- , CD11b+	Myeloid
Eosinophils	CD19- , CD3-, NK1.1-, F4/80- , Ly6C+ , Ly6G-	Myeloid
Total dendritic cells (DC)	CD19- , CD3-, NK1.1-, F4/80- , Ly6C- , Ly6G-	Myeloid
Plasmacytoid DC	CD19- , CD3-, NK1.1-, F4/80- , Ly6C+ , Ly6G- , CD317+	Myeloid
Conventional DC (cDC)	CD19- , CD3-, NK1.1-, F4/80- , Ly6G- , CD11c+ , MHCII+	Myeloid
CD11b type cDC	CD19- , CD3-, NK1.1-, F4/80- , Ly6G- , CD11c+ , MHCII+ , CD11b+	Myeloid
CD8a type cDC	CD19- , CD3-, NK1.1-, F4/80- , Ly6G- , CD11c+ , MHCII+ , CD11b-, CD86+	Myeloid
CD103+ CD8 type cDC	CD19- , CD3-, NK1.1-, F4/80- , Ly6G- , CD11c+ , MHCII+ , CD11b- ,CD86+, CD103+	Myeloid
